# Pathogenesis of Autoimmune Hepatitis—Cellular and Molecular Mechanisms

**DOI:** 10.3390/ijms222413578

**Published:** 2021-12-17

**Authors:** Claudia Sirbe, Gelu Simu, Iulia Szabo, Alina Grama, Tudor Lucian Pop

**Affiliations:** 1 2nd Pediatric Discipline, Department of Mother and Child, “Iuliu Hatieganu” University of Medicine and Pharmacy, 400012 Cluj-Napoca, Romania; claudia.sirbe@yahoo.com (C.S.); tudor.pop@umfcluj.ro (T.L.P.); 2 2nd Pediatric Clinic, Emergency Clinical Hospital for Children, 400177 Cluj-Napoca, Romania; 3 Cardiology Department, “Iuliu Hatieganu” University of Medicine and Pharmacy, 400012 Cluj-Napoca, Romania; simugelu@yahoo.com; 4 Cardiology Department, Rehabilitation Hospital, 400066 Cluj-Napoca, Romania; 5 Department of Rheumatology, “Iuliu Hatieganu” University of Medicine and Pharmacy, 400012 Cluj-Napoca, Romania; iulia_szabo90@yahoo.com

**Keywords:** autoimmune hepatitis, autoimmunity, genetic trait, pathogenesis, T cells, autoantibodies

## Abstract

Pediatric autoimmune liver disorders include autoimmune hepatitis (AIH), autoimmune sclerosing cholangitis (ASC), and de novo AIH after liver transplantation. AIH is an idiopathic disease characterized by immune-mediated hepatocyte injury associated with the destruction of liver cells, causing inflammation, liver failure, and fibrosis, typically associated with autoantibodies. The etiology of AIH is not entirely unraveled, but evidence supports an intricate interaction among genetic variants, environmental factors, and epigenetic modifications. The pathogenesis of AIH comprises the interaction between specific genetic traits and molecular mimicry for disease development, impaired immunoregulatory mechanisms, including CD4+ T cell population and Treg cells, alongside other contributory roles played by CD8+ cytotoxicity and autoantibody production by B cells. These findings delineate an intricate pathway that includes gene to gene and gene to environment interactions with various drugs, viral infections, and the complex microbiome. Epigenetics emphasizes gene expression through hereditary and reversible modifications of the chromatin architecture without interfering with the DNA sequence. These alterations comprise DNA methylation, histone transformations, and non-coding small (miRNA) and long (lncRNA) RNA transcriptions. The current first-line therapy comprises prednisolone plus azathioprine to induce clinical and biochemical remission. Further understanding of the cellular and molecular mechanisms encountered in AIH may depict their impact on clinical aspects, detect biomarkers, and guide toward novel, effective, and better-targeted therapies with fewer side effects.

## 1. Introduction

Pediatric autoimmune liver disorders include autoimmune hepatitis (AIH), autoimmune sclerosing cholangitis (ASC), and de novo AIH after liver transplantation [1]. AIH was first described in the 1950s [2]. Some reports suggest that the incidence of AIH in pediatric and in the general population has been rising in the last two decades [3,4], while others consider that these cases are more often diagnosed compared to the past because of increased disease awareness and the decreased number of cases of viral hepatitis after hepatitis B vaccination and hepatitis C effective treatment [1]. AIH has a female preponderance that is three times more frequent than in males [5]. The presence of autoimmunity in family is found in 40% of the cases, presenting overlapping autoimmune diseases, such as inflammatory bowel disease (IBD), nephrotic syndrome [6], thyroiditis [7], vitiligo, insulin-dependent diabetes [6], hemolytic anemia, idiopathic thrombocytopenia, celiac disease, and urticaria pigmentosa [7].

The broad clinical spectrum of pediatric AIH can range from an acute presentation with non-specific symptoms followed by jaundice, dark urine, and pale stools (in almost half of the patients with both types) [8,9], to severe acute hepatitis with liver failure developing encephalopathy in two weeks weeks–two months after presentation (3% of cases with AIH-1 and 25% of cases with AIH-2) [10,11]. There is also a description of a slowly progressive course of the disease that can last for a few months to a few years before diagnosis, characterized by malaise, headache, anorexia, weight loss, arthralgia, abdominal pain, and relapsing jaundice [1]. Only in rare asymptomatic cases is the diagnosis based on an incidental finding of modified laboratory investigations [12]. Approximately 10% of patients with both AIH types may present with end-stage liver disease and symptoms of portal hypertension, such as digestive bleeding and splenomegaly [13,14].

Some specific features can help establish the diagnosis of AIH: female preponderance [15], elevated immunoglobulin G (IgG), the presence of autoantibodies, and histological findings that suggest interface hepatitis [16,17]. The presence of antinuclear antibodies (ANA) and/or anti-smooth muscle antibodies (SMA) indicates AIH type 1 (AIH-1), while the presence of anti-liver kidney microsomal antibodies type one (LKM-1) and/or anti-liver cytosol type one antibodies (LC-1) are attributed to AIH type 2 (AIH-2) [18,19]. AIH-2 patients can associate partial IgA deficiency more often than AIH-1 patients [6,20].

AIH-1 is described in children and adults, while AIH-2 mainly affects children. There are little data based on the incidence of childhood AIH, but it is known that AIH-1 accounts for approximately 60% of cases and appears most commonly in adolescents, while AIH-2 appears more frequently in younger children and infants [5]. 

The diagnosis of AIH is based on clinical aspects, laboratory investigations comprising liver and immunology analyses and a liver biopsy. The International Autoimmune Hepatitis Group (IAIHG) proposed a diagnostic system that provides the probability of AIH using several positive and negative scores [21]. The simplified IAIHG criteria were suggested for being much easier to use in a clinical setting. The simplified score is based on IgG, autoantibodies, the histological examination, which form the positive criteria, and the exclusion of other causes of hepatitis, such as hepatitis B, C, or E virus, Wilson’s disease, or alcohol which form the negative criteria from the IAIHG score [22]. Neither scoring system is recommended to be used in juvenile AIH, especially in the presence of severe acute hepatitis [23]. Moreover, the cutoff value of autoantibodies is lower in pediatrics than in adults [24]. Many studies reported comparable performance parameters of the scoring systems [25], while one study demonstrated lower sensitivity for the 2007 simplified score than the 1999 revised score [26]. More recently, a Position Statement was published on the diagnosis and management of juvenile AIH by the ESPGHAN Hepatology Committee, also proposing a diagnostic score to help differentiate between AIH and ASC [1]. 

The current first-line therapy encompasses prednisolone plus azathioprine to induce clinical and biochemical remission [27]. Although most AIH patients show complete response to this therapy, some may rapidly progress to cirrhosis or liver failure due to poor response during the remission period or relapse after drug withdrawal [28]. Hence, it is necessary to research the pathogenesis of AIH and explore novel and effective therapies.

**Figure 1:**
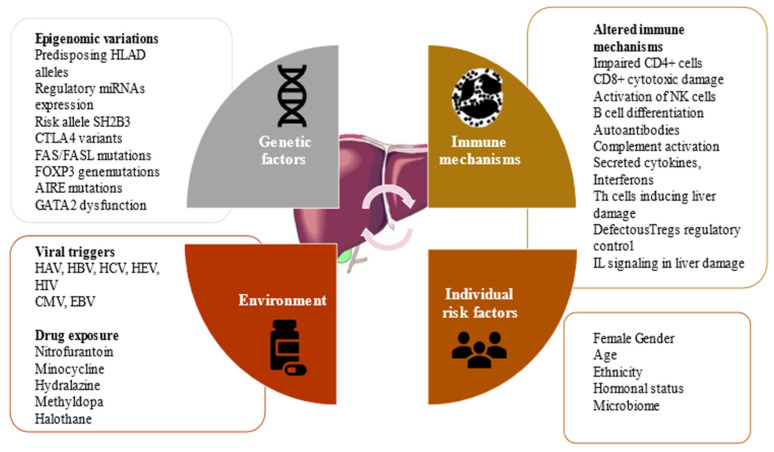
Predisposing factors associated with the risk of developing AIH. Abbreviations: AIRE, autoimmune regulator; CMV, cytomegalovirus; CTLA4, cytotoxic T lymphocyte antigen 4; EBV, Epstein–Barr virus; FAS/FASL, FOXP3, transcription factor forkhead box P3, GATA2, GATA-binding factor type 2; HAV, hepatitis A virus; HBV, hepatitis B virus; HCV, hepatitis C virus; HEV, hepatitis E virus; HIV, human immunodeficiency virus; HLA-D, human leukocyte antigen D allele; IL, interleukin; NK, natural killer cells; SH2B3, gene encoding adaptor protein also known as Lnk; Treg, regulatory T cell.

AIH pathophysiology is characterized by immune-mediated hepatocyte injury associated with the destruction of liver cells, causing inflammation, liver failure, and fibrosis. The etiology of AIH is not entirely unraveled [29,30], but evidence supports an intricate interaction among genetic variants, environmental factors, and epigenetic modifications [31]. The AIH-associated genetic predisposing loci shifted the interest of the scientific research community toward the contribution of epigenetics to disease development and its complex pathogenesis [32]. Predisposing factors associated with the risk of developing AIH are synthesized in Figure 1.

At the basis of AIH pathogenesis is the interaction between specific genetic traits and molecular mimicry for disease development, impaired immunoregulatory mechanisms, including CD4+ T cell population and Treg cells [33], alongside other contributory roles played by CD8+ cytotoxicity and autoantibody production by B cells [34]. These findings delineate an intricate pathway that includes gene to gene and gene to environment interactions with various drugs, viral infections, and the complex microbiome [32,35]. 

Epigenetics emphasizes gene expression through hereditary and reversible modifications of the chromatin architecture without interfering with the DNA sequence. These alterations comprise DNA methylation, histone transformations, and non-coding small (miRNA) and long (lncRNA) RNA transcriptions [36]. Epigenetic pathways intervene in various physiological mechanisms, such as cell division and differentiation and cell development and growth, and play an important role in various phenotypic features in health and disease [37]. The epigenome is prone to changes and can be modified by variable environmental factors, including infection, diet, medication, and chemicals [38,39,40]. 

DNA methylation is a process mediated by enzymes that occurs most often at the CpG sites where the location of cytosine is in the proximity of guanidine in the nucleotide sequence of the DNA structure [41]. DNA methyltransferases (DNMTs) generate 5-methylcytosine (5-mC) by the process of catalyzation with the inclusion of a methyl (CH3) group to the 5-carbon of the cytosine ring. The methylation status (5-mC content) of an array of CpG sites in the gene’s promoter region can influence gene transcription. This delineates into either silencing of the endogenous gene by methylation or enhancing gene transcription in diminished methylation [42]. Histone alterations encompass post-translational methylation, phosphorylation, acetylation, sumoylation, and ubiquitylation of histone proteins modifying the histone interaction with DNA molecules [43]. These changes result in the transformation of chromatin architecture, which can dictate if DNA is more or less accessible to genetic transcriptions, including enhancing or repressing gene transcription [44,45]. Non-coding RNAs (miRNAs) include less than 30 nucleotides, while lncRNAs contain more than 200 nucleotides. They intervene in gene expression at the transcriptional and post-transcriptional levels [46]. These RNA fragments are copies of gene sequences that are not transcribed into proteins. MiRNAs can bind co-transcriptional modification to an additional sequence from a targeted mRNA sequence and promote gene silencing. This is the result of mRNA translation or mRNA cleavage, based on the degree of complementarity [47]. MiRNAs have the capacity of controlling an array of mRNA targets, whereas the transcription of mRNA into proteins is modulated by a multitude of miRNAs [48]. 

The purpose of this review is to unveil the current status on the pathogenesis of AIH by focusing on cellular and molecular mechanisms and depicting their impact on clinical aspects, detecting biomarkers, and guiding toward novel, more effective, and better-targeted therapies.

Some of these factors are incompletely confirmed, such as CTLA4, FAS/FASL, AIRE, and GATA2 mutations, but the predisposing HLA-D allele, SH2B3 risk allele, female gender, age, hormonal status, and exposure to viral and drug triggers have been demonstrated to be associated with the risk of developing autoimmune hepatitis. 

## 2. Genetic Trait of Autoimmune Hepatitis

### 2.1. Human Leukocyte Antigen Associations

The mechanisms involved in autoimmune diseases represent a complex pathway between human leukocyte antigen (HLA) predisposing genes and non-HLA systems [32]. The genetic component of AIH is demonstrated in reports that studied monozygotic twins [49] and variations among different ethnicities, variety in the frequency, and age distribution of AIH among populations [50]. 

The genetic contribution to the AIH is based on over- and under-representation analyses of differentially expressed genes, genome-wide association studies (GWAS), which target genetic variants correlated with intricate traits, including the risk of developing AIH and the presumption that AIH is part of single abnormal genetic disorders. Preidentified HLA alleles are particularly informative for complex disease mapping and were first described in organ transplant compatibility. The specific alleles are encoded along HLA-A, -B, and -Cw regions on chromosome 6. These regions are associated with class I human major histocompatibility complex (MHC) and HLA-D regions (DR, DQ, and DP), which encode class II MHC. Class II MHC presents antigens to CD4+ (helper T cells), and CD8+ (cytotoxic T cells) is a component of the adaptive immune system response through apoptosis of recognized cells that present the antigen. To date, HLA-D regions are described in conjunction with the risk of developing AIH and other autoimmune diseases [50,51].

The importance of HLA variants implicated in AIH-1 was demonstrated by GWAS [52]. HLA genes responsible for AIH-1 susceptibility in adults are associated with the HLA-DRB1 variant on chromosome 6, representing a class II MHC. The HLA-DR3 (DRB1*0301) and -DR4 (DRB1*0401) molecules are described in North American and European populations [53,54] and also the -DR4 (DRB1*0401) allele in the Japanese population [55]. In a different study, patients that did not have HLA-DR3 or HLA-DR4 presented more often HLA-DR13 or HLA-DR7 and were indistinguishable from patients with HLA-DR3 regarding clinical and laboratory criteria. HLA-DR13 patients were younger than those with HLA-DR4 [56]. 

Studies based on the Argentinian population described HLA-DRB1*0405 in adults and HLA-DRB1*1301 and HLA-DRB1*0301 in children with AIH, with a protective effect of HLA-DRB*1302 [57]. In reports based on Mexican populations, DRB1*0404 and DQB1*0301 were over-represented in AIH patients [58]. Another report demonstrated that the association between AIH-1 and DRB1*0401 implies a less severe disease with a reduced risk of developing end-stage liver disease needing liver transplantation [59]. HLA genes responsible for AIH-1 susceptibility in children are associated with HLA-DR3 in Northern Europe, but the -DR4 encoding allele is not described as a genetic predisposition in pediatric AIH-1 [6]. There are multiple allele variants reported in AIH-1 susceptibility in different areas of the world, such as DRB1*08:02 and DRB1*08:03 in Japan, DRB1*04:05 in Korean populations, DRB1*13 and DRB1*14 in Pakistani populations [60], and DRB1*0405 and DRB1*1301, DQB1*02, and DQB1*0603 in Latin America [61]. In western India, DRB1*01 and DRB1*14 were associated with an increased risk of developing AIH-1 [62]. 

Genetic predispositions for AIH are reported worldwide for each area, but one study compared known susceptibility factors for AIH between patients from Italy and North America. This report showed an important difference in the genetic traits between the two cohorts, specifically the B8-DR3-DQ2 phenotype was more often described in Italian patients with AIH-1. At the same time, HLA-DR4, which is a significant risk factor for AIH in northern Europe and North America, was not encountered in the Italian cohort. Patients with AIH-1 from North America presented more frequently HLA-B8, -DR3 and HLA-DR4 than those from Italy. In addition, similar profiles were reported in both AIH-1 and AIH-2 in Italian patients: HLA-DR3, -DQ2 and -DR7. HLA-DR11 revealed a possible protective factor against AIH-1 in Italy. This study underlines different genetic backgrounds between two distinct countries, which suggests that other regional triggering events or other genetic factors may be involved that may influence the frequency of this disorder, clinical course, and treatment outcome [63]. 

AIH-2 is associated with predisposing DRB1*0701 [64] and DRB1*0301 [65]. Susceptibility for AIH-2 in Egypt is described in the genetic variant DRB1*15 [66]. In one quarter of the cases, the autoimmune polyendocrinopathy-candidiasis-ectodermal dystrophy (APECED) syndrome can be associated with AIH-2. The most common association is reported with HLA-DQB1*0301 and DQB1*0201 alleles [67]. In children with AIH-1 and AIH-2, a partial deficiency of the class III MHC complement component C4 was reported [68]. The HLA-DQ locus represents a predisposing genetic factor for AIH-2, while the HLA-DR locus is responsible for autoantibody development [69].

A murine model of AIH was generated by DNA immunization targeting AIH-2 self-antigens. The plasmid constructed for DNA immunization contained the extracellular region of a mouse cytotoxic T lymphocyte antigen 4 (CTLA-4) blended with the antigenic region of human CYP2D6 and formiminotransferase cyclodeaminase. Two peaks of increased ALT activity were described at four and seven months postinjection correlated with the development of antiLKM1 and antiLC1 autoantibodies and with liver inflammatory infiltrate containing CD4+ lymphocytes and CD8+ and B cells. This study showed that the break of tolerance against autoantigens triggered the development of AIH by molecular mimicry [70].

A Danish nationwide registry analysis demonstrated the intricate relation between genetic and environmental factors, showing that in families with cases of AIH, first-degree relatives present a fivefold risk of developing AIH [3]. The HLA-D regions intervene in the CD4+ T cell response to the CYP2D6 regions [71]. Antibodies to the soluble liver antigen or the liver/pancreas (anti SLA/LP) are associated with DRB1*0301. They have prognostic importance as they are present in severe forms of the disease with frequent relapse after therapy withdrawal [71,72]. 

The genetic architecture of autoimmune disorders encompasses complex traits, and the mechanism by which HLA regions confer susceptibility to developing autoimmunity is still undetermined [73]. One study mentioned that self-peptides in class II MHC could present an altered display at the thymic T cell selection, with a modified effector result and an impaired immune regulatory response [74], while class I MHC encoded by HLA-B regions could present an impaired function at some exogenous factors, such as abacavir and carbamazepine [71]. These described mechanisms comprise an important connection between the genetic architecture and environmental factors.

### 2.2. Non-Human Leukocyte Antigen

The GWAS is increasingly used to reveal the mechanisms of rare undiagnosed disorders, and specific ethnical genomes from various populations are especially important. This analysis helps identify the possible causal variants by verifying the particularities across populations. The study published in 2014 is the only study that reported the GWAS from patients with AIH-1 from the Netherlands and Germany. This report revealed an association between AIH and two susceptibility genotypes: HLA-DRB1*0301 and DRB1*0401 and also variants of SH2B3 (also known as SH2B adaptor protein 3 or as Lnk), which are associated with autoimmune diseases, such as AIH, type 1 diabetes mellitus, celiac disease, and rheumatoid arthritis [50]. Thus, the GWAS association with AIH inferred that the significance was lower than accepted, and SH2B3 should still be considered for its role in many autoimmune diseases. Non-synonymous single nucleotide polymorphism in SH2B3 is considered predisposing for various autoimmune and inflammatory diseases. SH2B3 encodes a common autoimmune locus; it regulates several cytokines signaling pathways and downregulates TNF (tumor necrosis factor), T cell activation, and Janus kinases 2 and 3 signaling. The Lnk variants in mice are linked to autoimmunity by elevated levels of activated T cells [72]. 

SH2B3 is also important in the mechanism of celiac disease by inflecting the immune response to gut bacteria. This is demonstrated by the presence of the SH2B3 rs3184504∗A risk allele in response to muramyl dipeptide and lipopolysaccharide and further indicates an augmented activation of the nucleotide-binding oligomerization domain 2 (NOD2). This risk allele and NOD2 pathway are also encountered in the pathogenesis of AIH and primary sclerosing cholangitis [75]. AIH is associated with the CTLA4 variants [76]. Some studies demonstrated the functional effect of vitamin D receptor variants in disease development, as these genotype variants are associated with variants in the fatty acid synthase (FAS) promoter or multifunctional pro-inflammatory cytokine that belongs to the TNF superfamily. This mechanism is suggested to result in the development of liver fibrosis [77,78].

The epigenetic component is influenced by changes in the histone methylation, microRNA (miRNA) profile, and mRNA translation into proteins [79]. MiRNAs are small endogenous RNA molecules of less than 30 nucleotides that regulate the transcription and translation of targeting RNAs based on the degree of complementarity [47]. Expression of miRNA is stable, reproducible, and persistent among members of the same species. MiRNAs are promising biomarkers, as their expression could reveal the information scattered on numerous target genes. [80]. Among these biomarkers is MiR-122, the most abundant miRNA in hepatocytes, involved in hepatitis C virus (HCV) replication, serving as a feasible therapeutic target [81]. The presence of MiR-122 is also mentioned in other liver diseases [82]. Reports demonstrate the importance of an array of regulatory miRNAs in disorders that comprise liver autoimmune and inflammation processes and metabolic syndromes [83]. MiRNAs control the innate and the adaptive immune response, and an error in miRNA expression has been correlated to human autoimmune diseases [84]. MiRNAs are emerging as important non-invasive diagnostic tools and can be helpful to predict the therapeutic response [85]. Here, we also reviewed various miRNA encountered in patients with AIH (Table 1). This unique expression of serum miRNAs can represent a non-invasive biomarker for AIH.

The importance of epigenomic variations in AIH is yet to be discovered. More research needs to be addressed to fully understand the role of the information encoded and the variability of translational modifications.

## 3. Monogenic Syndromes including Autoimmune Hepatitis

Various monogenic disorders are described, including AIH amongst other extrahepatic autoimmunity, underlining the importance of further research in these patterns of immune-mediated diseases.

### 3.1. Autoimmune Lymphoproliferative Syndrome and FAS/FAS Ligand

The autoimmune lymphoproliferative syndrome (ALPS) comprises autoimmune features with an elevated risk of malignancies. This chronic non-malignant lymphoproliferation includes hypergammaglobulinemia, autoimmune cytopenia, elevated IL-10 levels, FAS ligand, and the aggregation of double-negative T cells [92]. In addition, this disease can be associated with AIH type 2 [93]. 

This disease was first described in lpr and gld mice, which presented with FAS and FAS ligand mutations [94]. ALPS in human subjects present with either somatic or germline heterozygous FAS mutations or with both kinds of mutations [95]. FAS (or CD95, Apo1, and TNFRSF6) comprises the TNF receptor superfamily. Its role is mentioned in chronically activated lymphocytes apoptosis and blocking the self-reactive T and B lymphocytes [96]. The oligomerization of the FAS triggers an arrangement of a death-inducing signaling complex (DISC) which is augmented by the interaction between FAS and its ligand (CD178). The activation of DISC results in a caspase activation in permissive cells [97]. 

### 3.2. Regulatory T Cell Deficiency and Immunodysregulation Polyendocrinopathy Enteropathy X-Linked Syndrome

Some studies described multiple single-gene mutations responsible for the association between immunodeficiency and autoimmunity, including the presence of AIH, based on an impaired immune system. This category comprises the dysregulation of the immune system, polyendocrinopathy, enteropathy, and X-linked syndrome (IPEX). This is a rare, severe, and often fatal disease characterized by autoimmune enteropathy, thyroiditis, early-onset type 1 diabetes mellitus, and eczema [98,99]. The fundamental pathogenesis of the IPEX syndrome is the FOXP3 gene mutations. This gene encodes a DNA-binding protein, transcription factor forkhead box P3 (FOXP3), that is important for its role in inducing and maintaining peripheral immune tolerance. The FOXP3 protein contributes to the activation and differentiation of CD4^+^CD25^+^ regulatory T lymphocytes. The dysregulation of this pathway induces an error in immunologic reactivity and causes autoimmunity [100].

IPEX syndrome can also be associated with liver inflammation and AIH type 2 with LKM-1 antibodies [99]. Another study conducted on 173 cases with IPEX syndrome described the presence of hepatic abnormalities in 20% of the patients, most commonly with autoimmune hepatitis [101]. In addition, studies conducted on experimental animals demonstrated the development of systemic autoimmunity and AIH with liver lymphocytic infiltrate, elevated serum IgG, and antimitochondrial antibodies [102].

### 3.3. Cytotoxic T Lymphocyte Antigen 4 Mutations

The thymus offers partial protection against the formation of self-reactive T cells, but some self-reactive T cells can still spread in the body. In this process, an essential component is the cytotoxic T Lymphocyte Antigen 4 (CTLA-4, also known as CD152), which can control these cells. Studies on experimental mice reported that a CTLA-4 deficit can lead to immune dysregulation and autoimmunity [103,104]. Regulatory T cells (Treg cells) are the main cells expressing CTLA-4 [105]. Experimental studies demonstrated an analogous syndrome in CTLA-4-deficient mice to Treg cells deficiency [106]. CTLA-4 is encoded by the CTLA4 gene on chromosome 2 and directly antagonizes the co-stimulatory receptor CD28. CTLA-4 and CD28 have a similar structure and compete for activating ligands. The CTLA-4 deficit leads to stimulation of CD28 and its ligands CD80 and CD86, and this causes autoimmunity [107]. Studies reported that changes in CTLA-4 are associated with AIH, and a total deficit in mice leads to fatal autoimmunity with liver lymphocytic infiltrate [104].

CTLA-4 gene mutations in humans result in autosomal-dominant widespread autoimmunity, including AIH [108]. One study reported a case of de novo mutation of CTLA-4 with AIH that responded to treatment with abatacept [109]. Other studies noticed that drugs that block CTLA-4 such as ipilimumab used for cancer immunotherapy, deplete Treg cells and produce an AIH-like syndrome [110]. 

### 3.4. Autoimmune Regulator Mutations and the Autoimmune Polyendocrine Syndrome

The autoimmune-poly-endocrinopathy-candidiasis–ectodermal-dystrophy/dyspla-sia (APECED) or autoimmune polyendocrine syndrome type 1 represents a rare monogenic autosomal recessive disease, which is induced by mutations in the AIRE gene. The AIRE gene encodes the thymus-enriched transcription factor AIRE. It controls the central immune tolerance by deleting autoreactive T cells by negative selection and processing the autoantigens within the thymus [111,112]. The expression of the AIRE protein in thymic medullary epithelial cells and peripheral monocyte and dendritic cell lineage is for the variability of symptoms in APECED [113]. In addition, regarding APECED pathogenesis, a key contributor is a defect in the CD4+CD25+ regulatory T cells that normally intervene to prevent autoimmunity and peripheral tolerance [114]. Another factor influencing medullary thymic epithelial cell (mTEC) development is TRAF6, an E3 ubiquitin protein ligase. Mice with mTEC depletion of TRAF6 expression presented specific immunological and histological characteristics of human AIH, indicating that mTECs exert a central T cell tolerance and organ targeted autoimmunity but are unessential in peripheral tolerance [115]. 

APECED is characterized by immune-mediated Addison’s disease, hypoparathyroidism, and chronic mucocutaneous candidiasis [116]. The immune-mediated mucocutaneous candidiasis is induced by antibodies against the Th17/IL-17 pathway [117]. 

One study described APECED-associated hepatitis in almost half of the cases, but only a few patients presented the classical serological biomarkers, LKM-1 and SMA [118]. Immune-mediated AIH is induced by autoantibodies against CYP2D6 and CYP1A2 [119]. The mutations in the AIRE gene result in the development of AIH early in life and in AIH recurrence early after liver transplantation [120]. 

The fundamental nature of the defect in analogous AIRE mutations in mice results in a similar APECED syndrome accompanied by AIH, which is responsive to immunosuppressive therapy. AIH can also be treated with intact peripheral regulatory T cells, demonstrating a deficit in this population [121]. A recent study reported that a complementary system controls different antigens in the medullary thymic epithelium where the transcription factor FezF2 intervenes. The lack of this transcription factor in mice results in multisystem autoimmunity with a lymphocytic hepatic infiltrate, but with a different pattern from those with the AIRE mutation [122].

### 3.5. GATA-Binding Factor Type 2 Dysfunction

GATA2 is involved in the ontogenesis as a transcription factor of the hematopoietic system, hematopoietic stem cell activity, differentiation of myeloid and myelo-erythroid progenitor cell, and erythroid precursor cell maintenance [123]. GATA2 mutations were reported in patients with immunodeficiency syndromes, acute myeloid leukemia, and myelodysplastic syndrome [124]. One case was reported with GATA2 dysfunction associated with AIH and was responsive to immunosuppressive therapy [125]. 

## 4. Infectious and Environmental Triggers

### 4.1. Viral Triggers

Environmental factors such as viral infections are considered triggers for autoimmunity [126]. Hepatitis viruses can form neoantigens, resulting in the activation of autoreactive T cells and further generating inflammation [127]. 

Studies described the presence of similar autoantibodies in both AIH and viral hepatitis, especially hepatitis C virus (HCV). Molecular mimicry is reported between smooth muscle and viral antigens [128,129]. In support of this theory, it has been demonstrated that the laboratory tests presented false-positive antibodies for anti-HCV in untreated AIH with no evidence of HCV infection [130]. In HCV patients treated with high-dose interferon, induced autoimmunity was described in predisposed individuals who developed de novo non-hepatic disorders such as autoimmune thyroiditis. This association was not reported in cases with interferon-free HCV treatment [131]. Viral antigens were more likely to induce autoimmunity over time in HBV and HCV infections than in interferon treatment [132]. Patients with liver transplantation and interferon therapy for HCV administered before and after transplantation, had a higher risk of developing AIH, possibly triggered by immune-stimulating effects [133]. In children with chronic hepatitis B, ANA was found in 15% of the cases. Almost the same percentage of patients who were initially ANA negative were also reported with positive ANA during interferon treatment. Although interferon treatment was associated with the development of autoimmunity, this did not alter the treatment response [134]. 

Many studies described the association between AIH and other virus infections such as hepatitis A [135], the E virus [136,137], the Epstein–Barr virus [138], and cytomegalovirus [139]. In contrast, studies on large cohorts of patients showed no difference between the prevalence of viral hepatitis in relation to AIH and the general population [140,141]. Studies [142,143,144,145] demonstrated that infecting mice with adenovirus expressing human CYP2D6 generated persistent AIH in mice with liver necroinflammation and fibrosis. This marks the development of autoimmune liver disease after breaking immune tolerance in response to a viral infection expressing a human autoantigen [142]. The same infected mice were proposed as a model for revealing that only NOD genetic background could be susceptible for AIH induction but was still insufficient for generating autoimmunity spontaneously, marking the presence of strong environmental triggers [145]. The initial location of the autoantigen influenced different cell accumulation that induced inflammation, which affected hepatic stellate cell (HSC) activity, cell damage, and fibrosis [144].

### 4.2. Environmental Exposures

AIH has been linked to various drugs that can cause the appearance of autoantibodies to hepatocytes (Table 2), such as the inhalation of anesthetic halothane [146], dihydralazine [147], tienilic acid [148], nitrofurantoin, and minocycline. Autoantibodies are triggered by these chemical compounds, producing antibodies to CYP2C9 and developing hepatitis with positive ANA [149,150]. The hepatocyte surface antigen appears in the case of membrane alterations caused by halothane through the oxidative pathway. Observations of the interaction between circulating antibodies and the antigen were described in cases with severe liver necrosis after halothane anesthesia. This interaction is possible due to an altered membrane that provides the antigen composed by the oxidative route during anesthesia [151]. In experimental studies, another halogenated compound, trichloroethylene, could trigger antinuclear antibodies present in FAS mutant MRL+ mice, with lymphocytic hepatic infiltrate and lymphoproliferation [152]. Liver autoimmunity was also described in albendazole or isoniazid toxicity, where idiosyncratic liver injury or hypersensitivity are incriminated [153]. A difference should be made between drug-induced autoimmune injury (DILI) and drug-induced liver hepatitis, which requires immunosuppression for treatment and preventing relapses [154]. 

## 5. Specific Cell Types in AIH

The pathogenesis of AIH also involves an impairment of the effector and regulatory immunity (Table 3). In the immunoregulatory mechanisms, leukocytes present different composition in peripheral blood and in intrahepatic population regarding proportions and phenotypes (Table 4). Leukocytes involved in inflammation are targeted by immunosuppression therapy even before a certain diagnosis of AIH is established. This therapy usually comprises corticosteroids or antimetabolite agents. In this regard, it is frequently challenging to determine whether the results are attributed to the natural course of the disease or the administered medication [171].

### 5.1. CD4+ T Cells

CD4+ T cells (or helper) control B cell antibody production, influence CD8+ T cells cytotoxicity, regulate phagocytic processes and modulate movement within the cells [171]. CD4+ T cells are subclassified according to the cytokine they produce when stimulated. T helper 1 is associated with interferon-γ production, T helper 2 with IL-4 and IL-10, T helper 17 with IL-17, and regulatory T cells with IL-10. CD4+ can also suppress the activity of other T cells. The absence of CD4+ is important because it leads to profound immunodeficiency, being involved in various autoimmune disorders, and acquired immune deficiency syndrome (AIDS) [197,198]. Patients with AIH present impaired T cell number and function [126]. CD4+ important role is also represented by the presence of CD4+ in the inflammatory infiltrate of AIH [199]. 

There are multiple associations between HLA-D genotype variants and the development of AIH [50]. Class II MHC presents antigens to CD4+ and CD8+ and is part of the adaptive immune system response [50,51]. The impairment of these mechanisms leads to an autoimmune reaction with liver damage caused by interferon-γ released by effector T cells [200]. In experimental animal studies, stimulation of liver class II MHC is not enough to induce hepatitis [201]. 

The aforementioned monogenetic syndromes present pathways associated with CD4+ cells. AIH features imply the presence of CD4+ cells through IgG autoantibodies and interferon-γ production. Interestingly, in cases with infection caused by human immunodeficiency virus and treated with antiretroviral therapy, the association of AIH in immune reconstitution of CD4+ cells was observed [202]. Only one case was reported with the transfer of AIH by transplantation of disease-causing T cells [203]. 

The involvement of CD4+ cells in AIH pathogenesis is incompletely described. In this pathway, the importance of apoptosis inducted by CD95/FAS, and actions of interferon-γ, TNFα, and IL-17 are underlined. These markers are increased in the peripheral blood of AIH patients. In this regard, in monogenic diseases like ALPS, CD95+ cells with elevated expression of CD8+ and CD4+ are encountered [204]. Although this pathway is described in AIH, it is unclear if this is a specific feature for liver autoimmunity because it is also described in other liver diseases without the presence of autoimmunity [205]. 

The significant role of interferon in AIH is suggested by the surveillance of cases treated with exogenous interferons that develop AIH–like syndrome [149]. In this regard, experimental studies describe that hyperexpression of interferon-γ in mice produces features of AIH–like [160]. 

Th17 cells, another important pro-inflammatory factor in AIH [206], are overexpressed in both peripheral and intrahepatic populations [207]. Also, the Th17 response is influenced by IL-6 expression by hepatocytes. This observation is retained from studies on primary biliary cholangitis, which describe the association between the presence of Th17 cells and advanced disease [208,209]. The proinflammatory phenotype of AIH is attributed to the differentiation into Th17 instead of Treg cells, and therapy that targets IL-17 increases regulatory cells from CD4+CD25− cells derived from AIH patients [210]. Also, in this regard, in the resolution treatment of inflammation, Th17 cells are transdifferentiated into Treg cells [211]. 

### 5.2. Regulatory T Cells

Treg cells are a part of the CD4+ lymphocyte peripheral population with an important role in the innate and adaptive immune response by controlling the number and function of autoreactive T cells [212,213]. Their importance is underlined by the development of a severe form of autoimmune disease in their absence, such as the IPEX syndrome. Some studies described a decreased number of Treg cells in the inflammatory infiltrate of AIH [181] or in the peripheral blood when compared to other diseases [214,215]. More recently, improved molecular phenotyping with specific staining for factor FOXP3 measuring peripheral numbers and liver population of Treg cells has demonstrated a similar or increased number of Treg cells when compared to other liver disorders [216,217]. Patients with AIH also presented impaired T cell function regarding activation and proliferation of these cells [126]. Some reports described that this does not apply in the case of Treg cells isolated from liver tissue with inflammation [216,218]. 

A few studies described Treg cells as an important risk factor regarding the immune microenvironment in HCC development [219,220]. Most of the cases of HCC are encountered in HBV and HCV infection, and only a few cases are associated with AIH [219]. The increased percentage of CD4+CD25+ Treg cells in peripheral blood and in liver cells is thought to contribute to host immune response suppression during HBV and HCV infection [220,221,222,223], while AIH is related to numerical and functional Treg cells defect [181,224]. Findings regarding the HCC immune microenvironment mentioned Treg infiltration in chronic infections, while the precise pattern of Treg cells in AIH is not entirely known [225,226].

The functional impairment of peripheral Treg cells in AIH patients was demonstrated by reduced Treg cells responsiveness to IL-2, resulting in defective anti-Th1 cytokine IL-10 production [212]. Moreover, even in case of a decreased number, CD4+CD25+ T-cells of patients with AIH can maintain their regulatory function by reducing the number of interferon-γ-producing CD4+CD25- T-cells. If the central pathogenesis of AIH encounters the loss of immunoregulation, treatment should be focused on restoring Treg cells capacity to expand, with further increase of their number [200]. 

Children with AIH present a decreased number of Treg cells expressing CD39, leading to increased production of immunosuppressive adenosine. CD39+ Treg cells represent ectonucleotidase which controls extracellular nucleotide hydrolysis. The defect of this immunoregulatory mechanism causes a malfunction in proinflammatory nucleotides hydrolysis and overproduction of proinflammatory IL-17 produced by CD4+. The proinflammatory effect is caused by CD39+ Treg cells insufficient number, function, and increased transformation into CD4+ [227]. 

The decreased Treg cells favor disruption from the normal immunoregulatory mechanisms causing autoimmune liver disease with a proliferation of CD4+ and CD8+. Also, CD4+ are less sensitive to Treg cells regulatory control due to decreased expression of the inhibitory receptor T-cell-immunoglobulin-and-mucin-domain-containing-molecule-3 (Tim-3), which causes T lymphocyte effector death after ligation of galectin-9 controlled by Treg cells [228].

In AIH, CD4+CD127- T cells are impaired, which leads to Treg defects. These defects can be present in some cases even after treatment-induced remission [177].

The defective Treg cells lead to liver cells damage caused by an altered immune cascade consisting of cytotoxic T lymphocytes, activation of macrophages and complement, cytokines produced by Th1 and Th17 cells, increased adhesion of natural killer cells to a liver antibody Fc receptors ligation [229]. 

Analyzing the Treg cell function, the timing for obtaining samples unaffected by immunosuppression represents a problem. The main AIH treatment, corticosteroids and purine antimetabolites [230] have clear effects on Treg cell function [231,232,233,234]. 

Not all research studies agree with the contribution of Treg cells in the development of AIH. Some studies suggested that the number [214,235] and function of Treg cells are not impaired in AIH [214]. The decrease in Treg cells during therapy may be attributed to a decline in the Treg survival factor IL-2. This finding could contribute in the development of future treatment approaches [235]. Flow cytometry analysis and quantification of Treg-specific FOXP3 gene demethylation showed that the number of peripheral CD4+CD25+CD127- FOXP3+ Treg cells in AIH patients were not reduced compared to healthy subjects. However, Treg cells number was elevated in AIH cases with active disease compared to patients in disease remission, suggesting that the Treg cells number can be correlated with inflammation. The analysis of FOXP3 Treg cells on liver biopsy in AIH and NASH patients revealed that the intrahepatic Treg cells number was elevated in AIH patients compared to NASH patients, correlated with liver inflammation [214]. 

Another study suggested that Treg cells isolated from liver biopsy in patients with AIH were completely functional [216]. One theory mentioned the importance of the intrahepatic microenvironment suggesting that increased intrahepatic Treg cells could be caused by a homing of Treg cells into the inflamed liver [214]. Treg cell function requires the presence of IL-2, and in patients with AIH, the intrahepatic environment is deficient in IL-2 [216]. IL-2 is an essential cytokine for T cell survival and function, including Treg cells. Very low dose clinical grade IL-2 (VLDP, Proleukin) can generate STAT-5 phosphorylation, especially in peripheral and liver CD4+CD25+CD127- Treg cells in AIH patients. This is associated with phenotypic and functional transformations and also up-regulates anti-apoptotic protein Bcl-2 in Treg cells survival. The same study supports the potential VLDP treatment in AIH [236]. Regarding the number of Treg cells, the diminished number could be explained by specific sequestration of Treg cells into the intrahepatic environment with inflammation [237]. Furthermore, Treg cells can express CD40L and costimulatory markers when peripheral blood mononuclear cells are stimulated through complement receptors. In this way, the Treg population can influence dendritic cell functional maturation, suppresses CD4+ responses, and interact with B cells CD40 to generate Ig overproduction [238]. 

There are no studies describing antigen-specificity of the Treg population in human AIH. In experimental studies is mentioned that for autoimmunity to be controlled, antigen-specificity of Treg cells is required [239]. Similar work depicted the production of CYP2D6 antigen-specific Treg cells in AIH-2 patients [240]. This can also be demonstrated in animal models with APECED, where the transfer of functional Treg cells can alleviate disease [121]. 

Key factors surrounding the control of Treg cells over T lymphocyte responses include both CTLA4 and PD-1 (cell death protein-1) [241]. PD-1 action differs on Treg population versus CD4+, with an augmentation effect versus a proapoptotic effect. These findings delineate PD-1 blockade involvement in AIH development [164], while immune tolerance in liver transplant requires PD-1 expression [242]. Similar studies performed on animal models revealed that PD-1 insufficiency is correlated with AIH [243]. In order to clarify mechanisms involved in acute-onset fulminant AIH, one study developed AIH in a mouse model induced by loss of FOXP3 Treg cells and PD-1–mediated signaling which are responsible for regulating CD4+. Results demonstrated that in fatal AIH were involved dysregulated CD4+ from the spleen [244]. One study that analyzed Treg cells in treatment-naïve patients presented expression of memory cells suggesting previous antigen exposure, with decline in this proportion after therapy. Patients who did not respond to corticoid therapy presented decreased exhausted FOXP3^pos^ Treg cells and PD1 expression, which resulted in loss of CD4+ control with medication in these patients [245].

A future effective therapeutic approach could represent the usage of Treg cells in AIH patients, but the response to Treg infusions has not been demonstrated in large trials. One study showed that Treg cells from xenoimmunized mice expanded ex vivo could preserve their function and CXCR3 expression. These transferred Treg cells were recognized by the liver resembling an autologous transfer in AIH patients with induced remission [246]. One study demonstrated that intravenous infusion with good manufacturing practice (GMP)-grade autologous Treg cell cells in patients with AIH resulted in most of the homing to the liver and spleen with decreased migration to other organs. These transferred Treg cells showed increased survival in inflamed tissues, supporting Treg therapy for future clinical trials to prove the efficacy in AIH [247]. In experimental murine AIH treated with complexed IL-2/anti-IL-2, the mice showed elevation of intrahepatic and circulating Treg numbers after treatment and a decrease in activated, intrahepatic CD4+, restoring the immune balance and enhancing this approach for novel therapies [248]. When treatment with αCD3 monoclonal antibody, a subunit of the T cell receptor complex, was initiated in xenoimmunization mice, it prevented the development of AIH, and during active AIH, it decreased serum liver enzymes and autoantibody levels, and increased Tregs. These findings concluded that the usage of αCD3 antibody could be an effective treatment and should be considered for further testing in uncontrolled AIH [249].

### 5.3. CD8+ T Cells

CD8+ cells contribute to cell apoptosis with ligation through their T cell receptor to specific class I MHC molecule antigen. Cells that suffer apoptosis induced by CD8+ are damaged cells, tumoral, and virus-infected cells. Some of these cells can be encountered in the inflammatory liver tissue where CD8+ presents an augmented expression [250]. Combined studies on AIH report a diminished number of CD8+ compared to CD4+. However, CD8+ cells are the predominant population in interface lymphocytic infiltration in active AIH, while CD4+ cells are most often described in the central area of the portal tract [251]. Studies characterizing the critical factors present in AIH patients versus healthy individuals described upregulation of specific cell mediators of CD8+ cytotoxicity, such as perforin and granzyme B [252]. Combined, findings on both CD4+ and CD8+ delineate key factors in AIH-2 development, where antigen-specific CD8+ cells may be analyzed by MHC-I-tetramer staining. An association between CD8+ numbers and disease activity was described [253]. When studying animal models with AIH, the transfer of antigen-specific CD8+ will produce similar features to human disease [200]. One study generated bone marrow (bm) radiation chimeras which contained activated naïve transgenic CD8+ cells that were subject to co-stimulation by liver bm-derived cells. Results showed that proinflammatory cytokines (CD25 and CD54) are co-stimulation dependent, with differential T cell activation by hepatocytes and liver bm-derived cells. Donor CD8+ cells activated by liver bm-derived cells did not reveal detectable IL-2 level, with decreased function and increased pro-apoptotic factor Bim production [254]. 

### 5.4. γδ. T Cells

γδ T cells are T cells characterized by expression of heterodimeric T cell receptor containing γ and δ chains. Despite representing less than 5% of circulating lymphocytes, these cells are highly expressed in liver and intestinal mucosa [255]. Due to this important liver frequency, γδ cells have been studied in liver autoimmunity. γδ cells are thought to have a double role, both proinflammatory and anti-inflammatory activity. In this regard, an increased proportion of γδ cells was demonstrated in AIH, primary sclerosing cholangitis, and primary biliary cholangitis patients [182]. When comparing healthy individuals to AIH patients, an increased peripheral number of lymphocyte T cells was described with an inverted Vδ1: Vδ2 ratio corresponding to disease activity and a reverted ratio in correlation to disease remission [181]. Analysis of pediatric AIH cases delineated a high frequency of peripheral γδ cells compared to controls, with elevated expression of CD45RO in disease activity [256]. Similar studies conducted on adult cohorts with AIH concluded with a similar increased number of peripheral γδ cells with an inverted Vδ1: Vδ2 ratio. When comparing viral hepatitis and AIH patients, there was no difference in γδ cell levels [257]. One in vitro study on γδ cells isolated from liver biopsy mentioned hepatoma cell line cytotoxicity, but with no other tumor cytotoxicities [256]. The proper importance of γδ cells is yet to be discovered, but this cell population is also present in other autoimmune disorders such as Behcet’s disease and multiple sclerosis [258,259].

### 5.5. Natural Killer Cells

Natural killer (NK) cells are highly represented on liver histology compared to peripheral NK cells, presenting the important expression of inflammatory cytokines and cytotoxicity, but low-affinity Fc receptor CD16 expression [260,261]. Even if there are various debates regarding the role of NK cells in AIH, these cells are thought to contribute to fibrogenesis and tumor cell line control [262]. Experimental studies mentioned the role of NK cells in the development and control of T cell hepatitis. This was described when exposing liver cells to RNA analog and TLR3 ligand polyinosinic–polycytidylic acid (Poly I:C), which led to the development of NK cell-induced hepatitis with focal necrosis [263]. 

### 5.6. B Cells and Plasma Cells

The pathway of inflammatory diseases depicts the presence of plasma cells, a common feature also encountered in AIH [185]. Plasma cells result from activated B cells in the presence of CD4+ in the spleen or peripheral lymph nodes, which represent the secondary lymphoid tissue. There is also a possibility of tertiary lymphoid tissue described in intrahepatic inflammation when similar follicles are mentioned to those present in peripheral lymph nodes. This is defined as portal-associated lymphoid tissue [264]. Activation of B cell is required for specific autoantibody synthesis, which is the mark for human AIH. In this regard, the correlation between intraportal B cell population and blood IgG indicates autochthonous intrahepatic IgG synthesis [265]. Findings resulting from human AIH cohorts delineate key factors surrounding the significance of the B cell population in AIH pathogenesis. In support of this theory stands the efficiency of rituximab, a B cell–depleting CD20 antibody, in alleviating AIH [266,267]. When analyzing animal models, similar results were described. Mice coded with DNA for human liver antigens resulted in the appearance of hepatitis with lymphocytic infiltrate, which was mitigated after anti-CD20 administration [34]. Anti-CD20 treatment in patients with AIH and experimental murine AIH revealed that even with B cells and IgG reduction, monotherapy is not recommended as it can alter protein expression pattern triggering both inflammation and regeneration. In this regard, other immunotherapies should be considered for restoring immune tolerance in AIH patients [268]. A case study reported two AIH patients with compensated cirrhosis at diagnosis who did not respond to conventional treatment and received add-on B cell-activating factor (belimumab). Both patients presented a complete and persistent remission, indicating that belimumab could be an alternative treatment option for patients with treatment-refractory AIH and advanced fibrosis [269]. Even though some studies described B-cells defects in splenectomized patients [270,271], one study reported that splenectomized mice did not present alterations in CD8+ number and protective function [272]. 

### 5.7. Monocytes

Monocytes are generally not encountered in healthy liver but are a predominant component of inflammation in AIH [187]. Studies conducted on pediatric AIH cohorts mentioned that peripheral monocytes are highly represented in correlation to disease activity. In these cases, monocytes were more prone to migration, increased production of TNFα, and were less susceptible to an efficient control regarding the migration and IL-10 secretion promoted by Treg cells [224]. The comparison between peripheral and liver mononuclear cells concluded that the difference stands in the CD86 monocytes, which are diminished in the blood of the patients with AIH while being increased in the liver inflammatory infiltrate. This provides an insight into their role in T cell costimulation via CD86:CD28 interactions [273].

## 6. Unanswered Questions in Autoimmune Hepatitis

Despite the research performed over the past decades, the complete pathogenesis of AIH is only partially understood. Numerous studies suggested the implication of a susceptible genetic trait, in addition to various impaired immunity mechanisms. There are key factors that point toward a significant role of CD4+ in AIH pathways, but the consistent trigger for CD4+ activation is left unclear. There are contradictory results regarding Treg cell activity, but it is uncertain if a clear deficit can be attributed to disease development. Similarly, few functional studies described the precise mechanism of human B cells in AIH, but still, multiple questions remain unanswered. Another query is raised concerning the presence of autoantibodies in AIH about whether these can appear before the onset of disease, and when they appear if they are directly pathogenic. Also, an ambiguous explanation is bound around the fact that autoantibodies are associated with liver destruction even if some present broad extrahepatic binding. These findings delineate key factors surrounding AIH pathogenesis, combined with another trigger linked to environmental factors, such as hepatitis viruses. The specific role of environmental components is not fully understood, with further research being needed in this aspect. The genetic architecture of AIH with genome-wide variant association studies and sequencing studies is only at the starting point of a complete analysis, and further assessment techniques are developed to improve AIH management. The impact of epigenome and microbiome on AIH development is still at the onset of exploration.

## 7. Future Directions in Autoimmune Hepatitis Therapy

The treatment in AIH requires the selection of medication with fewer side effects on long-term immunosuppression and providing possible alternative treatment when the response is incomplete with first-line therapy. Future therapies should imply more specific treatment, with a clear difference between AIH and other autoimmune diseases, focusing precisely on AIH pathways. More descriptions of AIH pathogenesis should be documented to understand further how treatment choice should be addressed. Regarding the genetic architecture of AIH, there is only a single major GWAS for AIH-1, and there are no systematic studies for AIH-2. It should be of future concern to study genetic pathways that reveal molecular targets that can be rapidly exploited. AIH has well-known autoantigens, which can be used in antigen-specific therapy, such as peptide immunotherapy [274]. An ideal treatment in AIH should remove pathogenic autoimmune cells while carefully saving protective immunity, but actual strategies have proven elusive. An innovative therapeutic strategy would be one that can avoid general immunosuppression and can be applied in autoantibody-mediated diseases. This therapy is based on autoantigen chimeric immunoreceptors that can direct the T cell population to remove autoreactive B cell lymphocytes through B cell receptor specificity [275]. Lately, promising new therapies that can be used in autoimmune diseases are B-cell-directed treatments. These drugs block the B cell-activating factor (BAFF), which B cells require for normal development [276]. The questions raised dealt with the number or function of the Treg population in AIH generate uncertainty in a proper benefit of polyclonal or antigen-targeted Treg cell therapy [201]. In addition, another key factor that has been suggested is diet, especially fat consumption, which is supposed to affect the natural history of AIH [277]. A new revolutionary treatment could be extended from cancer checkpoint immunotherapy, with patients exposed to immune checkpoint inhibitors [278]. These findings delineate key factors surrounding AIH pathogenesis with more important genetic insights, which arise in support of individualized therapy.

## 8. Conclusions

Despite the research performed over the past decades, precipitating factors and pathogenetic regulatory pathways remain incompletely defined. The complete functional features in AIH are only partially understood. Reports suggest that at the basis of AIH development is an interaction between specific genetic traits and molecular mimicry for disease development and impairment of immunoregulatory mechanisms between effector and regulatory immunity with the CD4+ population and Treg cells, alongside the contributory roles played by CD8+ cytotoxicity and autoantibody production by the B cells. Furthermore, an important future research point comprises gene to gene and gene to environment interactions, with multiple targeting drugs, viral infections, and the complex microbiome. Similarly, experimental studies can offer discoveries on intricate combinations of defects in various pathways. Further research regarding the key factors in AIH etiopathogenesis will help provide a more profound understanding of novel and more individualized therapies.

## Figures and Tables

**Table 1:** Expression of miRNAs in AIH in human and animal cohorts.

Human miRNA	Upregulated	Reference	Downregulated	Reference
	miR-122-5p	[86]	miR-223-3p	[86]
	miR-1915-5p		miR-575	
	miR-193b-3p		miR-451a	
	miR-1908-3p		miR-4638-5p	
	miR-6073		miR-4443	
	miR-99a-5p		miR-486-5p	
	miR-602		miR-6765-3p	
	miR-1199-5p		miR-6820-5p	
	miR-1290		miR-4648	
	miR-21-5p		miR-6511a-5p	
	miR-4732-5p		miR-6889-5p	
	miR-122		miR-1207-5p	
	miR-192		miR-7150	
	miR-375		miR-6877-5p	
	miR-21		miR-4476	
			miR-6763-5p	
Animal miRNA				
	miR-10a, miR-133a	[87]	miR-15a/16-1	[88]
	miR-210	[89]	miR-155	[90]
	miR-155	[90]	miR-143-3p	[91]

**Table 2:** Drugs associated with the induction of AIH.

Drug	Comments	Selected References
Methyldopa	A possible toxic metabolic component that could present as an antigenic hapten on the surface of cells in susceptible hosts	[155,156]
Minocycline	Some associations with a rare HLA allele B*35:02, but most patients with similar clinical features lack this allele.	[149,157]
Nitrofurantoin	Not completely known. Drug metabolism produces oxidative free radicals, which can injure hepatocytes. Many cases are linked to HLA-DR6 and DR2 alleles.	[150,158]
α- and β-interferons	Immunomodulatory effects in presenting HLA antigens on hepatocyte surface and modifying CD4 and CD8+ T cell activity in predisposed patients. It can cause acute exacerbation of AIH and acute hepatitis-like syndrome that can coexist or be confused with chronic hepatitis B or C.	[133,159,160]
Hydralazine	Metabolized by N-acetyltransferase (NAT), more often associated with specific genetic variants in NAT activity, in the presence of autoantibodies to the P450 system (CYP 1A2).	[147,161]
Infliximab, adalimumab, etanercept	TNFα antagonists; the mechanism is not known, may induce and modulate autoimmunity.	[162,163]
Ipilimumab	AntiCTLA-4 inhibition and depletion of Treg cells	[110]
Nivolumab	PD-1 inhibition	[164]
Halothane	Partially modified by liver microsomal enzyme CYP 2E1 in trifluoroacetic acid. Halothane can trifluoroacetylate hepatic proteins, which can be immunogenic and produce cytotoxicity. In halothane hepatitis, antibodies to trifluoroacetylated proteins are present.	[165,166]
Tienilic acid (fenofibrate)	Not completely known. Liver immune reactivity may appear in the presence of altered metabolites or fenofibrate–protein haptens. Recognition of cytochrome P450 2C9 by antiLKM2 autoantibodies.	[167,168]
Non-steroidal anti-inflammatory drugs (diclofenac)	An immunoallergic component is linked to the genetic allele UGT 2B7, CYP 2C8, and ABC C2, genes being involved in the metabolism, conjugation, and excretion of diclofenac.	[169,170]

**Table 3:** Cellular mechanisms in autoimmune hepatitis.

Cell Type	Peripheral Blood			Liver		
	AIH vs. Healthy		Reference	AIH vs. Healthy		Reference
CD4+ T Cells	**↑**		[172]	↑		[172]
Th1 CD4+ T cells	NA		NA	↑		[173]
Th17 CD4+ T cells	↑		[174]	↑		[175]
CD4+CD25+ FOXP3 (Adults)	Number ↑	Function (-)	[176]	Number (-)	Function (-)	[176]
CD4+CD25+ (Adults and Children)	Number (-)	Function ↓	[177]	Number (-)	Function (-)	[177]
FOXP3 (Adults and Children)	Number ↓	Function (-)	[178]	Number (-)	Function (-)	[178]
CD8+ T cells	↑		[179]	↑		[180]
γδ T cells	↑		[181]	↑		[182]
Natural killer T cells			[181]	↑		[183]
B cells	=		[184]	=		[184]
Plasma cells	NA		NA	↑		[185]
Monocytes	↑		[186]	↑		[187]

AIH: autoimmune hepatitis; NA: not available; ↑: increased; ↓: decreased; (-): unknown data; =: no difference.

**Table 4:** Key inflammatory perturbations in autoimmune hepatitis.

Cell Type	Cell Stimuli	Secreted Cytokines	Peripheral Blood AIH vs. Healthy	Reference	Liver AIH vs. Healthy	Reference	Effects on Pathogenesis
Th1	IL-12	IL-2	↓	[188]	NA	NA	CD 8 (cytotoxic T cells) is a component of the adaptative immune system response through apoptosis of recognized cells on MHC class I and MHC class II on liver cells, which present the antigen Activation of NK cells
		IL-1ß	↑	[189]	↑	[190]	
		IFN-γ	↑	[191]	↑	[192]	
Th2	IL-4	IL-4	↓	[188]	↑	[183]	Promote CD4+ cells Promote B cell differentiation, plasma cells produce autoantibodies, and complement activation NK cells recognition of Fc receptor on hepatocyte surface IL-13 signaling in liver fibrogenesis Circulating IL-21 may predict the progression of necro-inflammatory activity on liver histology
		IL-10	↑	[172]	NA	NA	
		IL-13	↑	[190]	↑	[193]	
		IL-21	↑	[194]	NA	NA	
Th17	TGF-ß, IL-1ß, IL-6	IL-17	↑	[174]	↑	[174]	Elevated levels of IL-21 and IL-22 even in patients undergoing immunosuppressive therapy Th 17 cells induce liver damage and release of inflammation cytokines
		IL-22	↑	[195]	NA	NA	
		IL-23	↑	[195]	NA	NA	
		TNF-α	↑	[196]	↑	[192]	

AIH: autoimmune hepatitis; NA: not available; ↑: increased; ↓: decreased.

## References

[1] Mieli-Vergani G., Vergani D., Baumann U., Czubkowski P., Debray D., Dezsofi A., Fischler B., Gupte G., Hierro L., Indolfi G. (2018). Diagnosis and Management of Pediatric Autoimmune Liver Disease: ESPGHAN Hepatology Committee Position Statement. J. Pediatr. Gastroenterol. Nutr..

[2] Waldenstrom J. (1950). Leber, Blutproteine und Nahrungseiweiss. Dtsch. Gesellsch. Verd. Stoffw..

[3] Grønbæk L., Otete H., Ban L., Crooks C., Card T., Jepsen P., West J. (2020). Incidence, prevalence and mortality of autoimmune hepatitis in England 1997–2015. A population-based cohort study. Liver Int..

[4] Sebode M., Kloppenburg A., Aigner A., Lohse A.W., Schramm C., Linder R. (2020). Population-based study of autoimmune hepatitis and primary biliary cholangitis in Germany: Rising prevalences based on ICD codes, yet deficits in medical treatment. Z. Gastroenterol..

[5] Oettinger R., Brunnberg A., Gerner P., Wintermeyer P., Jenke A., Wirth S. (2005). Clinical features and biochemical data of Caucasian children at diagnosis of autoimmune hepatitis. J. Autoimmun..

[6] Gregorio G.V., Portmann B., Reid F., Donaldson P.T., Doherty D.G., McCartney M., Mowat A.P., Vergani D., Mieli-Vergani G. (1997). Autoimmune hepatitis in childhood: A 20-year experience. Hepatology.

[7] Wong G.-W., Yeong T., Lawrence D., Yeoman A.D., Verma S., Heneghan M.A. (2017). Concurrent extrahepatic autoimmunity in autoimmune hepatitis: Implications for diagnosis, clinical course and long-term outcomes. Liver Int..

[8] Jiménez-Rivera C., Ling S., Ahmed N., Yap J., Aglipay M., Barrowman N., Graitson S., Critch J., Rashid M., Ng V.L. (2015). Incidence and Characteristics of Autoimmune Hepatitis. Pediatrics.

[9] Yassin S., de Lacy R., Pillay K., Goddard E. (2020). Characteristics and Outcomes of Autoimmune Hepatitis from a Tertiary Paediatric Centre, Cape Town, South Africa. J. Trop. Pediatr..

[10] Di Giorgio A., Bravi M., Bonanomi E., Alessio G., Sonzogni A., Zen Y., Colledan M., D’Antiga L. (2015). Fulminant Hepatic Failure of Autoimmune Aetiology in Children. J. Pediatr. Gastroenterol. Nutr..

[11] Grama A., Aldea C.O., Burac L., Delean D., Bulata B., Sirbe C., Duca E., Boghitoiu D., Coroleuca A., Pop T.L. (2020). Etiology and Outcome of Acute Liver Failure in Children—The Experience of a Single Tertiary Care Hospital from Romania. Children.

[12] Brissos J., Carrusca C., Correia M., Cabral J. (2014). Autoimmune hepatitis: Trust in transaminases. BMJ Case Rep..

[13] Gregorio G.V., Portmann B., Karani J., Harrison P., Donaldson P.T., Vergani D., Mieli-Vergani G. (2001). Autoimmune Hepati-tis/Sclerosing Cholangitis Overlap Syndrome in Childhood: A 16-Year Prospective Study. Hepatology.

[14] Aljumah A.A., Al-Ashgar H., Fallatah H., Albenmousa A. (2019). Acute onset autoimmune hepatitis: Clinical presentation and treatment outcomes. Ann. Hepatol..

[15] Tenca A., Farkkila M., Jalanko H., Vapalahti K., Arola J., Jaakkola T., Penagini R., Vapalahti O., Kolho K.L. (2016). Environmental risk factors of pediatric-onset primary sclerosing cholangitis and autoimmune hepatitis. J. Pediatr. Gas Troenterol. Nutr..

[16] Zheng L., Liu Y., Shang Y., Han Z., Han Y. (2021). Clinical characteristics and treatment outcomes of acute severe autoimmune hepatitis. BMC Gastroenterol..

[17] Tanaka A. (2020). Autoimmune Hepatitis: 2019 Update. Gut Liver.

[18] Muratori P., Granito A., Quarneti C., Ferri S., Menichella R., Cassani F., Pappas G., Bianchi F.B., Lenzi M., Muratori L. (2009). Autoimmune hepatitis in Italy: The Bologna experience. J. Hepatol..

[19] Mack C.L., Adams D., Assis D.N., Kerkar N., Manns M.P., Mayo M.J., Vierling J.M., Alsawas M., Murad M.H., Czaja A.J. (2020). Diagnosis and Management of Autoimmune Hepatitis in Adults and Children: 2019 Practice Guidance and Guidelines from the American Association for the Study of Liver Diseases. Hepatology.

[20] Granito A., Pascolini S., Ricci C., Ferronato M., Muratori L., Vasuri F., Franceschini T., Lenzi M., Muratori P. (2021). Decompensated Cirrhosis as Presentation of LKM1/LC1 Positive Type 2 Autoimmune Hepatitis in Adulthood. A Rare Clinical Entity of Difficult Management. Gastroenterol. Insights.

[21] Alvarez F., Berg P., Bianchi F., Bianchi L., Burroughs A., Cancado E., Chapman R., Cooksley W., Czaja A., Desmet V. (1999). International Autoimmune Hepatitis Group Report: Review of criteria for diagnosis of autoimmune hepatitis. J. Hepatol..

[22] Hennes E.M., Zeniya M., Czaja A.J., Pares A., Dalekos G.N., Krawitt E.L., Bittencourt P.L., Porta G., Boberg K.M., Hofer H. (2008). Simplified criteria for the diagnosis of autoimmune hepatitis. Hepatology.

[23] Ferri P.M., Ferreira A.R., Miranda D.M., Silva A.C.S. (2012). Diagnostic criteria for autoimmune hepatitis in children: A challenge for pediatric hepatologists. World J. Gastroenterol..

[24] Vergani D., Alvarez F., Bianchi F.B., Cancado E., Mackay I.R., Manns M.P., Nishioka M., Penner E. (2004). Liver autoimmune serology: A consensus statement from the committee for autoimmune serology of the International Autoimmune Hepatitis Group. J. Hepatol..

[25] Czaja A.J. (2008). Performance parameters of the diagnostic scoring systems for autoimmune hepatitis. Hepatology.

[26] Niță A.F., Păcurar D. (2019). Adequacy of scoring systems in diagnosing paediatric autoimmune hepatitis: Retrospective study using a control group children with Hepatitis B infection. Acta Paediatr..

[27] Association for the Study of the Liver, European (2015). Corrigendum to ‘EASL Clinical Practice Guidelines: Autoimmune Hepatitis’. J. Hepatol..

[28] Pape S., Schramm C., Gevers T.J. (2019). Clinical management of autoimmune hepatitis. United Eur. Gastroenterol. J..

[29] Seldin M.F. (2015). The genetics of human autoimmune disease: A perspective on progress in the field and future directions. J. Autoimmun..

[30] Dywicki J., Buitrago-Molina L.E., Pietrek J., Lieber M., Broering R., Khera T., Schlue J., Manns M.P., Wedemeyer H., Jaeckel E. (2020). Autoimmune hepatitis induction can occur in the liver. Liver Int..

[31] Motawi T.K., El-Maraghy S.A., Sharaf S.A., Said S.E. (2019). Association of CARD10 rs6000782 and TNF rs1799724 variants with paediatric-onset autoimmune hepatitis. J. Adv. Res..

[32] Mells G.F., Kaser A., Karlsen T.H. (2013). Novel insights into autoimmune liver diseases provided by genome-wide association studies. J. Autoimmun..

[33] John K., Hardtke-Wolenski M., Jaeckel E., Manns M.P., Schulze-Osthoff K., Bantel H. (2017). Increased apoptosis of regulatory T cells in patients with active autoimmune hepatitis. Cell Death Dis..

[34] Béland K., Marceau G., Labardy A., Bourbonnais S., Alvarez F. (2015). Depletion of B cells induces remission of autoimmune hepatitis in mice through reduced antigen presentation and help to T cells. Hepatology.

[35] Wei Y., Li Y., Yan L., Sun C., Miao Q., Wang Q., Xiao X., Lian M., Li B., Chen Y. (2020). Alterations of gut microbiome in autoimmune hepatitis. Gut.

[36] Deng Q., Luo Y., Chang C., Wu H., Ding Y., Xiao R. (2019). The Emerging Epigenetic Role of CD8+T Cells in Autoimmune Diseases: A Systematic Review. Front. Immunol..

[37] Quintero-Ronderos P., Montoya-Ortiz G. (2012). Epigenetics and Autoimmune Diseases. Autoimmu. Dis..

[38] Toraño E.G., García M.G., Fernández-Morera J.L., Niño-García P., Fernández A.F. (2016). The Impact of External Factors on the Epigenome: In Utero and over Lifetime. BioMed Res. Int..

[39] Perera B., Faulk C., Svoboda L.K., Goodrich J.M., Dolinoy D.C. (2020). The role of environmental exposures and the epigenome in health and disease. Environ. Mol. Mutagen..

[40] Aguilera O., Fernández A.F., Muñoz A., Fraga M.F. (2010). Epigenetics and environment: A complex relationship. J. Appl. Physiol..

[41] Nan X., Meehan R.R., Bird A. (1993). Dissection of the methyl-CpG binding domain from the chromosomal protein MeCP2. Nucleic Acids Res..

[42] Ito S., Shen L., Dai Q., Wu S.C., Collins L.B., Swenberg J.A., He C., Zhang Y. (2011). Tet Proteins Can Convert 5-Methylcytosine to 5-Formylcytosine and 5-Carboxylcytosine. Science.

[43] Fuks F., Hurd P., Deplus R., Kouzarides T. (2003). The DNA methyltransferases associate with HP1 and the SUV39H1 histone methyltransferase. Nucleic Acids Res..

[44] Moore L.D., Le T., Fan G. (2013). DNA Methylation and Its Basic Function. Neuropsychopharmacology.

[45] Meda F., Folci M., Baccarelli A., Selmi C. (2011). The epigenetics of autoimmunity. Cell. Mol. Immunol..

[46] Treiber T., Treiber N., Meister G. (2012). Regulation of microRNA biogenesis and function. Thromb. Haemost..

[47] Bartel D.P. (2009). MicroRNAs: Target Recognition and Regulatory Functions. Cell.

[48] O’Brien J., Hayder H., Zayed Y., Peng C. (2018). Overview of MicroRNA Biogenesis, Mechanisms of Actions, and Circulation. Front. Endocrinol..

[49] Van Gerven N.M., Verwer B.J., Witte B.I., van Erpecum K.J., van Buuren H.R., Maijers I., Visscher A.P., Verschuren E.C., van Hoek B., Coenraad M.J. (2014). Epidemiology and clinical characteristics of autoimmune hepatitis in the Netherlands. Scand. J. Gastroenterol..

[50] Webb G., Hirschfield G. (2016). Using GWAS to identify genetic predisposition in hepatic autoimmunity. J. Autoimmun..

[51] Fernando M.M.A., Stevens C.R., Walsh E.C., de Jager P.L., Goyette P., Plenge R.M., Vyse T.J., Rioux J.D. (2008). Defining the Role of the MHC in Autoimmunity: A Review and Pooled Analysis. PLoS Genet..

[52] De Boer Y.S., van Gerven N.M., Zwiers A., Verwer B.J., van Hoek B., van Erpecum K.J., Beuers U., van Buuren H.R., Drenth J.P., Ouden J.W.D. (2014). Genome-Wide Association Study Identifies Variants Associated with Autoimmune Hepatitis Type 1. Gastroenterology.

[53] Donaldson P.T. (2002). Genetics in Autoimmune Hepatitis. Semin. Liver Dis..

[54] Van Gerven N.M., de Boer Y.S., Zwiers A., Verwer B.J., Drenth J.P., van Hoek B., van Erpecum K.J., Beuers U., van Buuren H.R., den Ouden J.W. (2015). Dutch Autoimmune Hepatitis Study Group. HLA-DRB1*03:01 and HLA-DRB1*04:01 modify the presentation and outcome in autoimmune hepatitis type-1. Genes Immun..

[55] Furumoto Y., Asano T., Sugita T., Abe H., Chuganji Y., Fujiki K., Sakata A., Aizawa Y. (2015). Evaluation of the role of HLA-DR antigens in Japanese type 1 autoimmune hepatitis. BMC Gastroenterol..

[56] Czaja A.J., Carpenter H.A., Moore S.B. (2006). Clinical and HLA phenotypes of type 1 autoimmune hepatitis in North American patients outside DR3 and DR4. Liver Int..

[57] Pando M.J., Larriba J., Fernandez G.C., Fainboim H., Ciocca M., Ramonet M., Badia I., Daruich J., Findor J., Tanno H. (1999). Pediatric and adult forms of type I autoimmune hepatitis in argentina: Evidence for differential genetic predisposition. Hepatology.

[58] Vázquez-García M.N., Aláez C., Olivo A., Debaz H., Pérez-Luque E., Burguete A., Cano S., de la Rosa G., Bautista N., Hernández A. (1998). MHC Class II Sequences of Suscepti-bility and Protection in Mexicans with Autoimmune Hepatitis. J. Hepatol..

[59] Kirstein M.M., Metzler F., Geiger E., Heinrich E., Hallensleben M., Manns M.P., Vogel A. (2015). Prediction of short- and long-term outcome in patients with autoimmune hepatitis. Hepatology.

[60] Higuchi T., Oka S., Furukawa H., Tohma S., Yatsuhashi H., Migita K. (2021). Genetic risk factors for autoimmune hepatitis: Implications for phenotypic heterogeneity and biomarkers for drug response. Hum. Genom..

[61] Duarte-Rey C., Pardo A.L., Rodríguez-Velosa Y., Mantilla R.D., Anaya J.-M., Rojas-Villarraga A. (2009). HLA class II association with autoimmune hepatitis in Latin America: A meta-analysis. Autoimmun. Rev..

[62] Shankarkumar U., Amarapurkar D.N., Kankonkar S. (2005). Human Leukocyte Antigen Allele Associations in Type-1 Autoimmune Hepatitis Patients from Western India. J. Gastroenterol. Hepatol..

[63] Muratori P., Czaja A.J., Muratori L., Pappas G., Maccariello S., Cassani F., Granito A., Ferrari R., Mantovani V., Lenzi M. (2005). Genetic distinctions between autoimmune hepatitis in Italy and North America. World J. Gastroenterol..

[64] Bittencourt P.L., Goldberg A.C., Cançado E.L., Porta G., Laudanna A.A., Kalil J. (1998). Different HLA profiles confer susceptibility to autoimmune hepatitis type 1 and 2. Am. J. Gastroenterol..

[65] Ma Y., Bogdanos D., Hussain M.J., Underhill J., Bansal S., Longhi M.S., Cheeseman P., Mieli–Vergani G., Vergani D. (2006). Polyclonal T-Cell Responses to Cytochrome P450IID6 Are Associated with Disease Activity in Autoimmune Hepatitis Type 2. Gastroenterology.

[66] Elfaramawy A.A., Elhossiny R.M., Abbas A.A., Aziz H.M. (2010). HLA-DRB1 as a risk factor in children with autoimmune hepatitis and its relation to hepatitis A infection. Ital. J. Pediatr..

[67] Meloni A., Willcox N., Meager A., Atzeni M., Wolff A.S.B., Husebye E.S., Furcas M., Rosatelli M.C., Cao A., Congia M. (2012). Autoimmune Polyendocrine Syndrome Type 1: An Extensive Longitudinal Study in Sardinian Patients. J. Clin. Endocrinol. Metab..

[68] Vergani D., Larcher V., Davies E., Wells L., Nasaruddin B., Mieli-Vergani G., Mowat A. (1985). Genetically Determined Low C4: A Predisposing Factor to Autoimmune Chronic Active Hepatitis. Lancet.

[69] Djilali-Saiah I., Fakhfakh A., Louafi H., Caillat-Zucman S., Debray D., Alvarez F. (2006). HLA class II influences humoral autoimmunity in patients with type 2 autoimmune hepatitis. J. Hepatol..

[70] Lapierre P., Djilali-Saiah I., Vitozzi S., Alvarez F. (2004). A murine model of type 2 autoimmune hepatitis: Xenoimmunization with human antigens. Hepatology.

[71] Illing P., Vivian J., Dudek N.L., Kostenko L., Chen Z., Bharadwaj M., Miles J., Kjer-Nielsen L., Gras S., Williamson N. (2012). Immune self-reactivity triggered by drug-modified HLA-peptide repertoire. Nat. Cell Biol..

[72] Katayama H., Mori T., Seki Y., Anraku M., Iseki M., Ikutani M., Iwasaki Y., Yoshida N., Takatsu K., Takaki S. (2014). Lnk prevents inflammatory CD8+T-cell proliferation and contributes to intestinal homeostasis. Eur. J. Immunol..

[73] Simmonds M.J., Gough S.C.L. (2007). The HLA Region and Autoimmune Disease: Associations and Mechanisms of Action. Curr. Genom..

[74] Grommé M., Neefjes J. (2002). Antigen degradation or presentation by MHC class I molecules via classical and non-classical pathways. Mol. Immunol..

[75] Zhernakova A., Elbers C.C., Ferwerda B., Romanos J., Trynka G., Dubois P.C., de Kovel C.G., Franke L., Oosting M., Barisani D. (2010). Evolutionary and Functional Analysis of Celiac Risk Loci Reveals SH2B3 as a Protective Factor against Bacterial Infection. Am. J. Hum. Genet..

[76] Agarwal K., Czaja A.J., Jones D.E., Donaldson P.T. (2000). Cytotoxic T lymphocyte antigen-4 (CTLA-4) gene polymorphisms and susceptibility to type 1 autoimmune hepatitis. Hepatology.

[77] Vogel A., Strassburg C.P., Manns M.P. (2002). Genetic association of vitamin D receptor polymorphisms with primary biliary cirrhosis and autoimmune hepatitis. Hepatology.

[78] Agarwal K., Czaja A.J., Donaldson P.T. (2007). A functional Fas promoter polymorphism is associated with a severe phenotype in type 1 autoimmune hepatitis characterized by early development of cirrhosis. Tissue Antigens.

[79] Lu Q. (2013). The critical importance of epigenetics in autoimmunity. J. Autoimmun..

[80] Schwarzenbach H., Nishida N., Calin G., Pantel K. (2014). Clinical relevance of circulating cell-free microRNAs in cancer. Nat. Rev. Clin. Oncol..

[81] Lanford R.E., Hildebrandt-Eriksen E.S., Petri A., Persson R., Lindow M., Munk M.E., Kauppinen S., Ørum H. (2010). Therapeutic silencing of microRNA-122 in primates with chronic hepatitis C virus infection. Science.

[82] Bandiera S., Pfeffer S., Baumert T.F., Zeisel M.B. (2015). MiR-122–A key factor and therapeutic target in liver disease. J. Hepatol..

[83] Szabo G., Bala S. (2013). MicroRNAs in liver disease. Nat. Rev. Gastroenterol. Hepatol..

[84] Zeng L., Cui J., Wu H., Lu Q. (2014). The emerging role of circulating microRNAs as biomarkers in autoimmune diseases. Autoimmunity.

[85] Wang X.W., Heegaard N.H.H., Ørum H. (2012). MicroRNAs in Liver Disease. Gastroenterology.

[86] Migita K., Komori A., Kozuru H., Jiuchi Y., Nakamura M., Yasunami M., Furukawa H., Abiru S., Yamasaki K., Nagaoka S. (2015). Circulating microRNA Profiles in Patients with Type-1 Autoimmune Hepatitis. PLoS ONE.

[87] Jia H.-Y., Chen F., Chen J.-Z., Wu S.-S., Wang J., Cao Q.-Y., Chen Z., Zhu H.-H. (2014). MicroRNA Expression Profiles Related to Early Stage Murine Concanavalin A-Induced Hepatitis. Cell. Physiol. Biochem..

[88] Lu Z., Liu J., Liu X., Huang E., Yang J., Qian J., Zhang D., Liu R., Chu Y. (2018). MicroRNA 15a/16-1 Suppresses Aryl Hydrocarbon Receptor-Dependent Interleukin-22 Secretion in CD4 + T Cells and Contributes to Immune-Mediated Organ Injury. Hepatology.

[89] Song G., Jia H., Xu H., Liu W., Zhu H., Li S., Shi J., Li Z., He J., Chen Z. (2013). Studying the association of microRNA-210 level with chronic hepatitis B progression. J. Viral Hepat..

[90] Xia G., Wu S., Wang X., Fu M. (2018). Inhibition of microRNA-155 attenuates concanavalin-A-induced autoimmune hepatitis by regulating Treg/Th17 cell differentiation. Can. J. Physiol. Pharmacol..

[91] Tu H., Chen D.-Z., Cai C., Du Q., Lin H., Pan T., Sheng L., Xu Y., Teng T., Tu J. (2020). Microrna-143-3p attenuated development of hepatic fibrosis in autoimmune hepatitis through regulation of TAK1 phosphorylation. J. Cell. Mol. Med..

[92] Worth A., Thrasher A., Gaspar H.B. (2006). Autoimmune lymphoproliferative syndrome: Molecular basis of disease and clinical phenotype. Br. J. Haematol..

[93] Pensati L., Costanzo A., Ianni A., Accapezzato D., Iorio R., Natoli G., Nisini R., Almerighi C., Balsano C., Vajro P. (1997). Fas/Apo1 mutations and autoimmune lymphoproliferative syndrome in a patient with type 2 autoimmune hepatitis. Gastroenterology.

[94] Nagata S., Suda T. (1995). Fas and Fas ligand: Lpr and gld mutations. Immunol. Today.

[95] Magerus-Chatinet A., Stolzenberg M.C., Lanzarotti N., Neven B., Daussy C., Picard C., Neveux N., Desai M., Rao M., Ghosh K. (2013). Autoimmune Lymphoproliferative Syndrome Caused by a Homo-zygous Null FAS Ligand (FASLG) Mutation. J. Allergy Clin. Immunol..

[96] Hao Z., Duncan G.S., Seagal J., Su Y.-W., Hong C., Haight J., Chen N.-J., Elia A., Wakeham A., Li W.Y. (2008). Fas Receptor Expression in Germinal-Center B Cells Is Essential for T and B Lymphocyte Homeostasis. Immunity.

[97] Strasser A., Jost P.J., Nagata S. (2009). The Many Roles of FAS Receptor Signaling in the Immune System. Immunity.

[98] Grimbacher B., Warnatz K., Yong P.F.K., Korganow A.S., Peter H.H. (2016). The Crossroads of Autoimmunity and Immunode-ficiency: Lessons from Polygenic Traits and Monogenic Defects. J. Allergy Clin. Immunol..

[99] López S.I., Ciocca M., Oleastro M., Cuarterolo M.L., Rocca A., de Dávila M.T., Roy A., Fernández M.C., Nievas E., Bosaleh A. (2011). Autoimmune Hepatitis Type 2 in a Child With IPEX Syndrome. J. Pediatr. Gastroenterol. Nutr..

[100] Wildin R.S., Smyk-Pearson S., Filipovich A.H. (2002). Clinical and molecular features of the immunodysregulation, polyendocrinopathy, enteropathy, X linked (IPEX) syndrome. J. Med. Genet..

[101] Gambineri E., Ciullini Mannurita S., Hagin D., Vignoli M., Anover-Sombke S., DeBoer S., Segundo G.R.S., Allenspach E.J., Favre C., Ochs H.D. (2018). Clinical, Immunological, and Molecular Heterogeneity of 173 Patients with the Phenotype of Immune Dysregulation, Polyendocrinopathy, Enteropathy, X-Linked (IPEX) Syndrome. Front. Immunol..

[102] Godfrey V.L., Wilkinson J.E., Russell L.B. (1991). X-linked lymphoreticular disease in the scurfy (sf) mutant mouse. Am. J. Pathol..

[103] Waterhouse P., Penninger J.M., Timms E., Wakeham A., Shahinian A., Lee K.P., Thompson C.B., Griesser H., Mak T.W. (1995). Lymphoproliferative Disorders with Early Lethality in Mice Deficient in Ctla-4. Science.

[104] Tivol E.A., Borriello F., Schweitzer A., Lynch W.P., Bluestone J.A., Sharpe A. (1995). Loss of CTLA-4 leads to massive lymphoproliferation and fatal multiorgan tissue destruction, revealing a critical negative regulatory role of CTLA-4. Immunity.

[105] Takahashi T., Tagami T., Yamazaki S., Uede T., Shimizu J., Sakaguchi N., Mak T.W., Sakaguchi S. (2000). Immunologic Self-Tolerance Maintained by Cd25+Cd4+Regulatory T Cells Constitutively Expressing Cytotoxic T Lymphocyte–Associated Antigen 4. J. Exp. Med..

[106] Klocke K., Sakaguchi S., Holmdahl R., Wing K. (2016). Induction of autoimmune disease by deletion of CTLA-4 in mice in adulthood. Proc. Natl. Acad. Sci. USA.

[107] Tai X., van Laethem F., Sharpe A.H., Singer A. (2007). Induction of Autoimmune Disease in CTLA-4-/- Mice Depends on a Specific CD28 Motif That Is Required for In Vivo Costimulation. Proc. Natl. Acad. Sci. USA.

[108] Schubert D., Bode C., Kenefeck R., Hou T.Z., Wing J.B., Kennedy A., Bulashevska A., Petersen B.-S., Schäffer A.A., Grüning B. (2014). Autosomal dominant immune dysregulation syndrome in humans with CTLA4 mutations. Nat. Med..

[109] Lee S., Moon J.S., Lee C.-R., Kim H.-E., Baek S.-M., Hwang S., Kang G.H., Seo J.K., Shin C.H., Kang H.J. (2016). Abatacept alleviates severe autoimmune symptoms in a patient carrying a de novo variant in CTLA-4. J. Allergy Clin. Immunol..

[110] Kim K.W., Ramaiya N.H., Krajewski K.M., Jagannathan J.P., Tirumani S.H., Srivastava A., Ibrahim N. (2013). Ipilimumab associated hepatitis: Imaging and clinicopathologic findings. Investig. New Drugs.

[111] Fierabracci A. (2016). Type 1 Diabetes in Autoimmune Polyendocrinopathy-Candidiasis-Ectodermal Dystrophy Syndrome (APECED): A “Rare” Manifestation in a “Rare” Disease. Int. J. Mol. Sci..

[112] Assis D.N. (2020). Immunopathogenesis of Autoimmune Hepatitis. Clin. Liver Dis..

[113] Suzuki E., Kobayashi Y., Kawano O., Endo K., Haneda H., Yukiue H., Sasaki H., Yano M., Maeda M., Fujii Y. (2008). Expression of AIRE in Thymocytes and Peripheral Lymphocytes. Autoimmunity.

[114] Ryan K.R., Lawson C.A., Lorenzi A.R., Arkwright P.D., Isaacs J.D., Lilic D. (2005). CD4+CD25+ T-Regulatory Cells Are Decreased in Patients with Autoimmune Polyendocrinopathy Candidiasis Ectodermal Dystrophy. J. Allergy Clin. Immunol..

[115] Bonito A.J., Aloman C., Fiel M.I., Danzl N.M., Cha S., Weinstein E.G., Jeong S., Choi Y., Walsh M.C., Alexandropoulos K. (2013). Medullary thymic epithelial cell depletion leads to autoimmune hepatitis. J. Clin. Investig..

[116] Ahonen P., Myllärniemi S., Sipilä I., Perheentupa J. (1990). Clinical Variation of Autoimmune Polyendocrinopathy-Candidiasis-Ectodermal Dystrophy (APECED) in a Series of 68 Patients. N. Engl. J. Med..

[117] Kisand K., Wolff A.S.B., Podkrajšek K.T., Tserel L., Link M., Kisand K.V., Ersvaer E., Perheentupa J., Erichsen M.M., Bratanic N. (2010). Chronic mucocutaneous candidiasis in APECED or thymoma patients correlates with autoimmunity to Th17-associated cytokines. J. Exp. Med..

[118] Chascsa D.M., Ferré E.M.N., Hadjiyannis Y., Alao H., Natarajan M., Quinones M., Kleiner D.E., Simcox T.L., Chitsaz E., Rose S.R. (2021). APECED-Associated Hepatitis: Clinical, Biochemical, Histological and Treatment Data From a Large, Predominantly American Cohort. Hepatology.

[119] Obermayer–Straub P., Perheentupa J., Braun S., Kayser A., Barut A., Loges S., Harms A., Dalekos G., Strassburg C.P., Manns M.P. (2001). Hepatic autoantigens in patients with autoimmune polyendocrinopathy-candidiasis-ectodermal dystrophy. Gastroenterology.

[120] Lankisch T.O., Strassburg C.P., Debray D., Manns M.P., Jacquemin E. (2005). Detection of Autoimmune Regulator Gene Mutations in Children with Type 2 Autoimmune Hepatitis and Extrahepatic Immune-Mediated Diseases. J. Pediatr..

[121] Hardtke-Wolenski M., Taubert R., Noyan F., Sievers M., Dywicki J., Schlue J., Falk C.S., Lundgren B.A., Scott H.S., Pich A. (2015). Autoimmune hepatitis in a murine autoimmune polyendocrine syndrome type 1 model is directed against multiple autoantigens. Hepatology.

[122] Takaba H., Morishita Y., Tomofuji Y., Danks L., Nitta T., Komatsu N., Kodama T., Takayanagi H. (2015). Fezf2 Orchestrates a Thymic Program of Self-Antigen Expression for Immune Tolerance. Cell.

[123] Tsai F.-Y., Keller G., Kuo F.C., Weiss M., Chen J., Rosenblatt M., Alt F.W., Orkin S.H. (1994). An early haematopoietic defect in mice lacking the transcription factor GATA-2. Nat. Cell Biol..

[124] Hsu A.P., Sampaio E.P., Khan J., Calvo K.R., Lemieux J.E., Patel S.Y., Frucht D.M., Vinh D.C., Auth R.D., Freeman A.F. (2011). Mutations in GATA2 Are Associated with the Autosomal Dominant and Sporadic Monocytopenia and Mycobacterial Infection (MonoMAC) Syn-drome. Blood.

[125] Webb G., Chen Y.-Y., Li K.-K., Neil D., Oo Y., Richter A., Bigley V., Collin M., Adams D., Hirschfield G.M. (2016). Single-gene association between GATA-2 and autoimmune hepatitis: A novel genetic insight highlighting immunologic pathways to disease. J. Hepatol..

[126] Floreani A., Restrepo P., Secchi M.F., de Martin S., Leung P.S., Krawitt E., Bowlus C., Gershwin M.E., Anaya J.M. (2018). Etiopathogenesis of autoimmune hepatitis. J. Autoimmun..

[127] Fujinami R.S., von Herrath M.G., Christen U., Whitton J.L. (2006). Molecular Mimicry, Bystander Activation, or Viral Persistence: Infections and Autoimmune Disease. Clin. Microbiol. Rev..

[128] Gregorio G.V., Choudhuri K., Ma Y., Pensati P., Iorio R., Grant P., Garson J., Bogdanos D., Vegnente A., Mieli-Vergani G. (2003). Mimicry between the hepatitis C virus polyprotein and antigenic targets of nuclear and smooth muscle antibodies in chronic hepatitis C virus infection. Clin. Exp. Immunol..

[129] Vegnente G., Mieli-Vergani D., Vergani G.V., Gregorio K., Choudhuri Y., Ma A. (1999). Chronic Hepatitis B Virus Infection Nuclear and Smooth Muscle Antibodies in Polymerase and the Antigenic Targets of Mimicry Between the Hepatitis B Virus DNA. J. Immunol. Ref..

[130] Nishiguchi S., Kuroki T., Ueda T., Fukuda K., Takeda T., Nakajima S., Shiomi S., Kobayashi K., Otani S., Hayashi N. (1992). Detection of Hepatitis C Virus Antibody in the Absence of Viral RNA in Patients with Autoimmune Hepatitis. Ann. Intern. Med..

[131] Fattovich G., Giustina G., Favarato S., Ruol A. (1996). A survey of adverse events in 11,241 patients with chronic viral hepatitis treated with alfa interferon. J. Hepatol..

[132] Păcurar D., Dijmărescu I., Dijmărescu A., Pavelescu M., Andronie M., Becheanu C. (2019). Autoimmune phenomena in treated and naive pediatric patients with chronic viral hepatitis. Exp. Ther. Med..

[133] García-Buey L., García-Monzón C., Rodriguez S., Borque M.J., García-Sánchez A., Iglesias R., DeCastro M., Mateos F.G., Vicario J., Balas A. (1995). Latent autoimmune hepatitis triggered during interferon therapy in patients with chronic hepatitis C. Gastroenterology.

[134] Pop T.L., Stefănescu A., Samaşca G., Miu N. (2014). Clinical Significance of the Antinuclear Antibodies in Chronic Viral Hepatitis B in Children. Clin. Lab..

[135] Skoog S.M., Rivard R.E., Batts K.P., Smith C.I. (2002). Autoimmune Hepatitis Preceded by Acute Hepatitis A Infection. Am. J. Gastroenterol..

[136] Nagasaki F., Ueno Y., Mano Y., Igarashi T., Yahagi K., Niitsuma H., Okamoto H., Shimosegawa T. (2005). A Patient with Clinical Features of Acute Hepatitis E Viral Infection and Autoimmune Hepatitis. Tohoku J. Exp. Med..

[137] Pischke S., Gisa A., Suneetha P.V., Wiegand S.B., Taubert R., Schlue J., Wursthorn K., Bantel H., Raupach R., Bremer B. (2014). Increased HEV Seroprevalence in Patients with Autoimmune Hepatitis. PLoS ONE.

[138] Vento S., Guella L., Mirandola F., Cainelli F., Di Perri G., Solbiati M., Concia E., Ferraro T. (1995). Epstein-Barr virus as a trigger for autoimmune hepatitis in susceptible individuals. Lancet.

[139] Yokomori H.H., Toyoda-Akui M., Kaneko F., Shimizu Y., Takeuchi H., Tahara K., Yoshida H., Kondo H., Motoori T., Ohbu M. (2011). Association of an overlap syndrome of autoimmune hepatitis and primary biliary cirrhosis with cytomegalovirus infection. Int. J. Gen. Med..

[140] Lohse A.W., Gerken G., Mohr H., Löhr H.F., Treichel U., Dienes H.P., Büschenfelde K.H.M.Z. (1995). Relation between autoimmune liver diseases and viral hepatitis: Clinical and serological characteristics in 859 patients. Z. Gastroenterol..

[141] Van Gerven N.M., van der Eijk A.A., Pas S.D., Zaaijer H.L., de Boer Y.S., Witte B.I., van Nieuwkerk C.M., Mulder C.J., Bouma G., de Man R.A. (2016). Dutch Autoimmune Hepatitis Study Group. Seroprevalence of Hepatitis E Virus in Autoimmune Hepatitis Patients in the Netherlands. J. Gastrointestin. Liver Dis..

[142] Holdener M., Hintermann E., Bayer M., Rhode A., Rodrigo E., Hintereder G., Johnson E.F., Gonzalez F.J., Pfeilschifter J., Manns M.P. (2008). Breaking tolerance to the natural human liver autoantigen cytochrome P450 2D6 by virus infection. J. Exp. Med..

[143] Hintermann E., Ehser J., Christen U. (2012). The CYP2D6 Animal Model: How to Induce Autoimmune Hepatitis in Mice. J. Vis. Exp..

[144] Hintermann E., Ehser J., Bayer M., Pfeilschifter J.M., Christen U. (2013). Mechanism of autoimmune hepatic fibrogenesis induced by an adenovirus encoding the human liver autoantigen cytochrome P450 2D6. J. Autoimmun..

[145] Hardtke-Wolenski M., Fischer K., Noyan F., Schlue J., Falk C.S., Stahlhut M., Woller N., Kuehnel F., Taubert R., Manns M.P. (2013). Genetic predisposition and environmental danger signals initiate chronic autoimmune hepatitis driven by CD4+T cells. Hepatology.

[146] Vergani D., Mieli-Vergani G., Alberti A., Neuberger J., Eddleston A.L.W.F., Davis M., Williams R. (1980). Antibodies to the Surface of Halothane-Altered Rabbit Hepatocytes in Patients with Severe Halothane-Associated Hepatitis. N. Engl. J. Med..

[147] Bourdi M., Tinel M., Beaune P.H., Pessayre D. (1994). Interactions of dihydralazine with cytochromes P4501A: A possible expla-nation for the appearance of anti-cytochrome P4501A2 autoantibodies. Mol. Pharmacol..

[148] Kurth M.J., Yokoi T., Gershwin M.E. (2014). Halothane-induced hepatitis: Paradigm or paradox for drug-induced liver injury. Hepatology.

[149] Gough A., Chapman S., Wagstaff K., Emery P., Elias E. (1996). Minocycline induced autoimmune hepatitis and systemic lupus erythematosus-like syndrome. BMJ.

[150] Appleyard S., Saraswati R., A Gorard D. (2010). Autoimmune hepatitis triggered by nitrofurantoin: A case series. J. Med. Case Rep..

[151] Neuberger J., Mieli-Vergani G., Tredger J.M., Davis M., Williams R. (1981). Oxidative metabolism of halothane in the production of altered hepatocyte membrane antigens in acute halothane-induced hepatic necrosis. Gut.

[152] Griffin J.M., Gilbert K.M., Lamps L.W., Pumford N.R. (2000). CD4(+) T-Cell Activation and Induction of Autoimmune Hepatitis Following Trichloroethylene Treatment in MRL+/+ Mice. Toxicol. Sci..

[153] Grama A., Aldea C., Burac L., Delean D., Boghitoiu D., Bulata B., Nitescu V., Ulmeanu C., Pop T.L. (2022). Acute liver failure secondary to toxic exposure in children. Arch. Med. Sci..

[154] Björnsson E., Talwalkar J., Treeprasertsuk S., Kamath P.S., Takahashi N., Sanderson S., Neuhauser M., Lindor K. (2010). Drug-induced autoimmune hepatitis: Clinical characteristics and prognosis. Hepatology.

[155] (2020). Methyldopa. LiverTox: Clinical and Research Information on Drug-Induced Liver Injury. https://www.ncbi.nlm.nih.gov/books/NBK548173/.

[156] Rodman J.S., Deutsch D.J., Gutman S.I. (1976). Methyldopa Hepatitis. A Report of Six Cases and Review of the Literature. Am. J. Med..

[157] (2019). Minocycline. LiverTox: Clinical and Research Information on Drug-Induced Liver Injury. https://www.ncbi.nlm.nih.gov/books/NBK547956/.

[158] (2020). Nitrofurantoin. LiverTox: Clinical and Research Information on Drug-Induced Liver Injury. https://www.ncbi.nlm.nih.gov/books/NBK548318/.

[159] (2021). Alpha Interferon-LiverTox-NCBI Bookshelf. https://www.ncbi.nlm.nih.gov/books/NBK547867/.

[160] Berardi S., Lodato F., Gramenzi A., D’Errico A., Lenzi M., Bontadini A., Morelli M.C., Tamè M.R., Piscaglia F., Biselli M. (2007). High Incidence of Allograft Dysfunction in Liver Transplanted Patients Treated with Pegylated-Interferon Alpha-2b and Ribavirin for Hepatitis C Recurrence: Possible de Novo Autoimmune Hepatitis?. Gut.

[161] (2018). Hydralazine. LiverTox: Clinical and Research Information on Drug-Induced Liver Injury. https://www.ncbi.nlm.nih.gov/books/NBK548580/.

[162] (2019). Autoimmune Hepatitis. LiverTox: Clinical and Research Information on Drug-Induced Liver Injury. https://www.ncbi.nlm.nih.gov/books/NBK548188/.

[163] Germano V., Diamanti A.P., Baccano G., Natale E., Muda A.O., Priori R., Valesini G. (2005). Autoimmune hepatitis associated with infliximab in a patient with psoriatic arthritis. Ann. Rheum. Dis..

[164] Michot J., Bigenwald C., Champiat S., Collins M., Carbonnel F., Postel-Vinay S., Berdelou A., Varga A., Bahleda R., Hollebecque A. (2016). Immune-related adverse events with immune checkpoint blockade: A comprehensive review. Eur. J. Cancer.

[165] (2018). Halothane. LiverTox: Clinical and Research Information on Drug-Induced Liver Injury. https://www.ncbi.nlm.nih.gov/books/NBK548151/.

[166] Gut J., Christen U., Frey N., Koch V., Stoffler D. (1995). Molecular mimicry in halothane hepatitis: Biochemical and structural characterization of lipoylated autoantigens. Toxicology.

[167] (2017). Fenofibrate. LiverTox: Clinical and Research Information on Drug-Induced Liver Injury. https://www.ncbi.nlm.nih.gov/books/NBK548607/.

[168] Lecoeur S., Bonierbale E., Challine D., Gautier J.-C., Valadon P., Dansette P., Catinot R., Ballet F., Mansuy D., Beaune P.H. (1994). Specificity of In Vitro Covalent Binding of Tienilic Acid Metabolites to Human Liver Microsomes in Relationship to the Type of Hepatotoxicity: Comparison with Two Directly Hepatotoxic Drugs. Chem. Res. Toxicol..

[169] Scully L.J., Clarke D., Barr R.J. (1993). Diclofenac Induced Hepatitis. 3 Cases with Features of Autoimmune Chronic Active Hepatitis. Dig. Dis. Sci..

[170] (2021). Diclofenac-LiverTox-NCBI Bookshelf. https://www.ncbi.nlm.nih.gov/books/NBK547953/.

[171] Norris S., Collins C., Doherty D., Smith F., McEntee G., Traynor O., Nolan N., Hegarty J., O’Farrelly C. (1998). Resident human hepatitis lymphocytes are phenotypically different from circulating lymphocytes. J. Hepatol..

[172] Löhr H.F., Schlaak J.F., Gerken G., Fleischer B., Dienes H.-P., Büschenfelde K.-H.M.Z. (1994). Phenotypical analysis and cytokine release of liver-infiltrating and peripheral blood T lymphocytes from patients with chronic hepatitis of different etiology. Liver Int..

[173] Löhr H., Manns M., Kyriatsoulis A., Lohse A.W., Trautwein C., Büschenfelde K.-H.M.Z., Fleischer B. (1991). Clonal analysis of liver-infiltrating T cells in patients with LKM-1 antibody-positive autoimmune chronic active hepatitis. Clin. Exp. Immunol..

[174] Zhao L., Tang Y., You Z., Wang Q., Liang S., Han X., Qiu D., Wei J., Liu Y., Shen L. (2011). Interleukin-17 contributes to the pathogenesis of autoimmune hepatitis through inducing hepatic interleukin-6 ex-pression. PLoS ONE.

[175] Oo Y.H., Adams D.H. (2012). Regulatory T cells and autoimmune hepatitis: Defective cells or a hostile environment?. J. Hepatol..

[176] Hu E.-D., Chen D.-Z., Wu J.-L., Lu F.-B., Chen L., Zheng M.-H., Li H., Huang Y., Li J., Jin X.-Y. (2018). High fiber dietary and sodium butyrate attenuate experimental autoimmune hepatitis through regulation of immune regulatory cells and intestinal barrier. Cell. Immunol..

[177] Longhi M.S., Ma Y., Mitry R.R., Bogdanos D., Heneghan M., Cheeseman P., Mieli-Vergani G., Vergani D. (2005). Effect of CD4+CD25+ regulatory T-cells on CD8 T-cell function in patients with autoimmune hepatitis. J. Autoimmun..

[178] Longhi M.S., Hussain M.J., Mitry R.R., Arora S.K., Mieli-Vergani G., Vergani D., Ma Y. (2006). Functional Study of CD4+CD25+ Regulatory T Cells in Health and Autoimmune Hepatitis. J. Immunol..

[179] Suzuki Y., Kobayashi M., Hosaka T., Someya T., Akuta N., Kobayashi M., Suzuki F., Tsubota A., Saitoh S., Arase Y. (2004). Peripheral CD8+/CD25+ Lymphocytes May Be Implicated in Hepatocellular Injuries in Patients with Acute-Onset Autoimmune Hepatitis. J. Gastroenterol..

[180] Eggink H.F., Houthoff H.J., Huitema S., Gips C.H., Poppema S. (1982). Cellular and humoral immune reactions in chronic active liver disease. I. Lymphocyte subsets in liver biopsies of patients with untreated idiopathic autoimmune hepatitis, chronic active hepatitis B and primary biliary cirrhosis. Clin. Exp. Immunol..

[181] Ferri S., Longhi M.S., de Molo C., Lalanne C., Muratori P., Granito A., Hussain M.J., Ma Y., Lenzi M., Mieli-Vergani G. (2010). A multifaceted imbalance of T cells with regulatory function characterizes type 1 autoimmune hepatitis. Hepatology.

[182] Martins E.B., Graham A.K., Chapman R.W., Fleming K.A. (1996). Elevation of Gamma Delta T Lymphocytes in Peripheral Blood and Livers of Patients with Primary Sclerosing Cholangitis and Other Autoimmune Liver Diseases. Hepatology.

[183] Cherñavsky A.C., Paladino N., Rubio A.E., de Biasio M.B., Periolo N., Cuarterolo M., Goñi J., Galoppo C., Cañero-Velasco M.C., Muñoz A.E. (2004). Simultaneous expression of th1 cytokines and IL-4 confers severe characteristics to type I autoimmune hepatitis in children. Hum. Immunol..

[184] Webb G., Hirschfield G., Krawitt E., Gershwin M. (2018). Cellular and Molecular Mechanisms of Autoimmune Hepatitis. Annu. Rev. Pathol. Mech. Dis..

[185] Czaja A.J., Carpenter H.A. (1993). Sensitivity, specificity, and predictability of biopsy interpretations in chronic hepatitis. Gastroenterology.

[186] Senaldi G., Portmann B., Mowat A.P., Mieli-Vergani G., Vergani D. (1992). Immunohistochemical features of the portal tract mononuclear cell infiltrate in chronic aggressive hepatitis. Arch. Dis. Child..

[187] Huang R., Wu H., Liu Y., Yang C., Pan Z., Xia J., Xiong Y., Wang G., Sun Z., Chen J. (2016). Increase of infiltrating monocytes in the livers of patients with chronic liver diseases. Discov. Med..

[188] Czaja A.J., Sievers C., Zein N.N. (2000). Nature and Behavior of Serum Cytokines in Type 1 Autoimmune Hepatitis. Dig. Dis. Sci..

[189] Luan J., Zhang X., Wang S., Li Y., Fan J., Chen W., Zai W., Wang S., Wang Y., Chen M. (2018). NOD-Like Receptor Protein 3 Inflammasome-Dependent IL-1β Accelerated ConA-Induced Hepatitis. Front. Immunol..

[190] Noel G., Arshad M.I., Filliol A., Genet V., Rauch M., Lucas-Clerc C., Lehuen A., Girard J.P., Piquet-Pellorce C., Samson M. (2016). Ablation of Interaction between IL-33 and ST2+ Regulatory T Cells Increases Immune Cell-Mediated Hepatitis and Activated NK Cell Liver Infiltration. Am. J. Physiol. Gastrointest. Liver Physiol..

[191] Landi A., Weismuller T.J., Lankisch T.O., Santer D.M., Tyrrell D.L.J., Manns M.P., Houghton M. (2014). Differential Serum Levels of Eosinophilic Eotaxins in Primary Sclerosing Cholangitis, Primary Biliary Cirrhosis, and Autoimmune Hepatitis. J. Interf. Cytokine Res..

[192] Hussain M.J., Mustafa A., Gallati H., Mowat A.P., Mieli-Vergani G., Vergani D. (1994). Cellular Expression of Tumour Necrosis Factor-Alpha and Interferon-Gamma in the Liver Biopsies of Children with Chronic Liver Disease. J. Hepatol..

[193] Tacke F., Hammerich L. (2014). Interleukins in chronic liver disease: Lessons learned from experimental mouse models. Clin. Exp. Gastroenterol..

[194] Abe K., Takahashi A., Imaizumi H., Hayashi M., Okai K., Kanno Y., Watanabe H., Ohira H. (2016). Interleukin-21 plays a critical role in the pathogenesis and severity of type I autoimmune hepatitis. SpringerPlus.

[195] Thomas-Dupont P., Remes-Troche J.M., Izaguirre-Hernández I.Y., Vargas L.A.S., Maldonado-Rentería M.D.J., Hernández-Flores K.G., Torre A., Bravo-Sarmiento E., Vivanco-Cid H. (2016). Elevated circulating levels of IL-21 and IL-22 define a cytokine signature profile in type 2 autoimmune hepatitis patients. Ann. Hepatol..

[196] Maggiore G., de Benedetti F., Massa M., Pignatti P., Martini A. (1995). Circulating Levels of Interleukin-6, Interleukin-8, and Tumor Necrosis Factor-Alpha in Children with Autoimmune Hepatitis. J. Pediatr. Gastroenterol. Nutr..

[197] Zhu J., Paul W.E. (2008). CD4 T cells: Fates, functions, and faults. Blood.

[198] Palmer M.T., Weaver C.T. (2009). Autoimmunity: Increasing suspects in the CD4+ T cell lineup. Nat. Immunol..

[199] Wen L., Peakman M., Lobo-Yeo A., Vergani D., Mowat A., Vergani M., McFarlane B. (1990). T-cell-directed hepatocyte damage in autoimmune chronic active hepatitis. Lancet.

[200] Longhi M.S., Ma Y., Bogdanos D.P., Cheeseman P., Mieli-Vergani G., Vergani D. (2004). Impairment of CD4(+)CD25(+) Regulatory T-Cells in Autoimmune Liver Disease. J. Hepatol..

[201] Herkel J., Jagemann B., Wiegard C., Lazaro J.F.G., Lüth S., Kanzler S., Blessing M., Schmitt E., Lohse A.W. (2003). MHC class II-expressing hepatocytes function as antigen-presenting cells and activate specific CD4 T lymphocyutes. Hepatology.

[202] O’Leary J.G., Zachary K., Misdraji J., Chung R.T. (2008). De Novo Autoimmune Hepatitis during Immune Reconstitution in an HIV-Infected Patient Receiving Highly Active Antiretroviral Therapy. Clin. Infect. Dis..

[203] Granito A., Stanzani M., Muratori L., Bogdanos D.P., Muratori P., Pappas G., Quarneti C., Lenzi M., Vergani D., Bianchi F.B. (2008). LKM1-Positive Type 2 Autoimmune Hepatitis Following Allogenic Hematopoietic Stem-Cell Transplantation. Am. J. Gastroenterol..

[204] Ogawa S., Sakaguchi K., Takaki A., Shiraga K., Sawayama T., Mouri H., Miyashita M., Koide N., Tsuji T. (2000). Increase in CD95 (Fas/APO-1)-positive CD4+and CD8+T cells in peripheral blood derived from patients with autoimmune hepatitis or chronic hepatitis C with autoimmune phenomena. J. Gastroenterol. Hepatol..

[205] Zwolak A., Surdacka A., Daniluk J. (2016). Bcl-2 and Fas expression in peripheral blood leukocytes of patients with alcoholic and autoimmune liver disorders. Hum. Exp. Toxicol..

[206] Buitrago-Molina L.E., Dywicki J., Noyan F., Trippler M., Pietrek J., Schlue J., Manns M.P., Wedemeyer H., Jaeckel E. (2021). Splenectomy Prior to Experimental Induction of Autoimmune Hepatitis Promotes More Severe Hepatic Inflammation, Production of IL-17 and Apoptosis. Biomedicines.

[207] An J. (2019). Expression and Significance of Th17 Cells and Related Factors in Patients with Autoimmune Hepatitis. Comb. Chem. High Throughput Screen..

[208] Lan R.Y., Salunga T.L., Tsuneyama K., Lian Z.-X., Yang G.-X., Hsu W., Moritoki Y., Ansari A.A., Kemper C., Price J. (2009). Hepatic IL-17 responses in human and murine primary biliary cirrhosis. J. Autoimmun..

[209] Yang C.Y., Ma X., Tsuneyama K., Huang S., Takahashi T., Chalasani N.P., Bowlus C.L., Yang G.X., Leung P.S., Ansari A.A. (2014). IL-12/Th1 and IL-23/Th17 Biliary Microenvironment in Primary Biliary Cirrhosis: Implications for Therapy. Hepatology.

[210] Longhi M.S., Liberal R., Holder B., Robson S.C., Ma Y., Mieli–Vergani G., Vergani D. (2012). Inhibition of Interleukin-17 Promotes Differentiation of CD25 Cells Into Stable T Regulatory Cells in Patients With Autoimmune Hepatitis. Gastroenterology.

[211] Gagliani N., Amezcua Vesely M.C., Iseppon A., Brockmann L., Xu H., Palm N.W., de Zoete M.R., Licona-Limón P., Paiva R.S., Ching T. (2015). Th17 cells transdifferentiate into regulatory T cells during resolution of inflammation. Nature.

[212] Liberal R., Grant C.R., Holder B.S., Cardone J., Martinez-Llordella M., Ma Y., Heneghan M.A., Mieli-Vergani G., Vergani D., Longhi M.S. (2015). In autoimmune hepatitis type 1 or the autoimmune hepatitis-sclerosing cholangitis variant defective regu-latory T-cell responsiveness to IL-2 results in low IL-10 production and impaired suppression. Hepatology.

[213] Wang H., Feng X., Yan W., Tian D. (2020). Regulatory T Cells in Autoimmune Hepatitis: Unveiling Their Roles in Mouse Models and Patients. Front. Immunol..

[214] Peiseler M., Sebode M., Franke B., Wortmann F., Schwinge D., Quaas A., Baron U., Olek S., Wiegard C., Lohse A.W. (2012). FOXP3+ regulatory T cells in autoimmune hepatitis are fully functional and not reduced in frequency. J. Hepatol..

[215] Behairy B.E., El-Araby H.A., Abd El kader H.H., Ehsan N.A., Salem M.E., Zakaria H.M., Khedr M.A. (2017). Assessment of Intrahepatic Regulatory T Cells in Children with Autoimmune Hepatitis. Ann. Hepatol..

[216] Chen Y., Jeffery H.C., Hunter S., Bhogal R., Birtwistle J., Braitch M.K., Roberts S., Ming M., Hannah J., Thomas C. (2016). Human intrahepatic regulatory T cells are functional, require IL-2 from effector cells for survival, and are susceptible to Fas ligand-mediated apoptosis. Hepatology.

[217] Oo Y.H., Sakaguchi S. (2013). Regulatory T-cell directed therapies in liver diseases. J. Hepatol..

[218] Granito A., Muratori P., Ferri S., Pappas G., Quarneti C., Lenzi M., Bianchi F.B., Muratori L. (2009). Diagnosis and therapy of autoimmune hepatitis. Mini Rev. Med. Chem..

[219] Global Burden of Disease Liver Cancer Collaboration (2017). The Burden of Primary Liver Cancer and Underlying Etiologies From 1990 to 2015 at the Global, Regional, and National Level: Results From the Global Burden of Disease Study 2015. JAMA Oncol..

[220] Xu D., Fu J., Jin L., Zhang H., Zhou C., Zou Z., Zhao J.-M., Zhang B., Shi M., Ding X. (2006). Circulating and Liver Resident CD4+CD25+ Regulatory T Cells Actively Influence the Antiviral Immune Response and Disease Progression in Patients with Hepatitis B. J. Immunol..

[221] Stoop J.N., van der Molen R.G., Baan C., van der Laan L., Kuipers E.J., Kusters J.G., Janssen H.L.A. (2005). Regulatory T cells contribute to the impaired immune response in patients with chronic hepatitis B virus infection. Hepatology.

[222] Yang G., Liu A., Xie Q., Guo T.B., Wan B., Zhou B., Zhang J.Z. (2006). Association of CD4+CD25+Foxp3+ regulatory T cells with chronic activity and viral clearance in patients with hepatitis B. Int. Immunol..

[223] Peng G., Li S., Wu W., Sun Z., Chen Y., Chen Z. (2008). Circulating CD4+ CD25+ regulatory T cells correlate with chronic hepatitis B infection. Immunology.

[224] Longhi M.S., Meda F., Wang P., Samyn M., Mieli-Vergani G., Vergani D., Ma Y. (2008). Expansion and de novo generation of potentially therapeutic regulatory T cells in patients with autoimmune hepatitis. Hepatology.

[225] Kobayashi N., Hiraoka N., Yamagami W., Ojima H., Kanai Y., Kosuge T., Nakajima A., Hirohashi S. (2007). FOXP3+ Regulatory T Cells Affect the Development and Progression of Hepatocarcinogenesis. Clin. Cancer Res..

[226] Kurebayashi Y., Kubota N., Sakamoto M. (2021). Immune microenvironment of hepatocellular carcinoma, intrahepatic cholangiocarcinoma and liver metastasis of colorectal adenocarcinoma: Relationship with histopathological and molecular classifications. Hepatol. Res..

[227] Grant C.R., Liberal R., Holder B.S., Cardone J., Ma Y., Robson S.C., Mieli-Vergani G., Vergani D., Longhi M.S. (2014). Dysfunctional CD39(POS) Regulatory T Cells and Aberrant Control of T-Helper Type 17 Cells in Autoimmune Hepatitis. Hepatology.

[228] Liberal R., Grant C.R., Holder B.S., Ma Y., Mieli-Vergani G., Vergani D., Longhi M.S. (2012). The Impaired Immune Regulation of Autoimmune Hepatitis Is Linked to a Defective Galectin-9/Tim-3 Pathway. Hepatology.

[229] Manns M.P., Griffin K.J., Sullivan K., Johnson E.F. (1991). LKM-1 autoantibodies recognize a short linear sequence in P450IID6, a cytochrome P-450 monooxygenase. J. Clin. Investig..

[230] Pape S., Nevens F., Verslype C., Mertens C., Drenth J.P., Tjwa E.T. (2020). Profiling the patient with autoimmune hepatitis on calcineurin inhibitors: A real-world-experience. Eur. J. Gastroenterol. Hepatol..

[231] Daniel V., Trojan K., Opelz G. (2016). Immunosuppressive drugs affect induction of IFNy+ Treg in vitro. Hum. Immunol..

[232] Akimova T., Kamath B.M., Goebel J.W., Meyers K.E.C., Rand E.B., Hawkins A., Levine M., Bucuvalas J.C., Hancock W.W. (2012). Differing Effects of Rapamycin or Calcineurin Inhibitor on T-Regulatory Cells in Pediatric Liver and Kidney Transplant Recipients. Arab. Archaeol. Epigr..

[233] Vierling J.M., Kerkar N., Czaja A.J., Mack C.L., Adams D., Assis D.N., Manns M.P., Mayo M.J., Nayfeh T., Majzoub A.M.M. (2020). Immunosuppressive Treatment Regimens in Auto-immune Hepatitis: Systematic Reviews and Meta-Analyses Supporting American Association for the Study of Liver Diseases Guidelines. Hepatology.

[234] Raffin C., Vo L.T., Bluestone J.A. (2020). T Reg Cell-Based Therapies: Challenges and Perspectives. Nature Reviews. Immunology.

[235] Diestelhorst J., Junge N., Schlue J., Falk C.S., Manns M.P., Baumann U., Jaeckel E., Taubert R. (2017). Pediatric autoimmune hepatitis shows a disproportionate decline of regulatory T cells in the liver and of IL-2 in the blood of patients undergoing therapy. PLoS ONE.

[236] Jeffery H.C., Jeffery L.E., Lutz P., Corrigan M., Webb G.J., Hirschfield G.M., Adams D.H., Oo Y.H. (2017). Low-Dose Interleu-kin-2 Promotes STAT-5 Phosphorylation, T Reg Survival and CTLA-4-Dependent Function in Autoimmune Liver Diseases. Clin. Exp. Immunol..

[237] Oo Y.H., Banz V., Kavanagh D., Liaskou E., Withers D., Humphreys E., Reynolds G.M., Lee-Turner L., Kalia N., Hubscher S.G. (2012). CXCR3-dependent recruitment and CCR6-mediated positioning of Th-17 cells in the inflamed liver. J. Hepatol..

[238] Tedesco D., Thapa M., Gumber S., Elrod E.J., Rahman K., Ibegbu C.C., Magliocca J.F., Adams A.B., Anania F., Grakoui A. (2017). CD4+ Foxp3+ T Cells Promote Aberrant IgG Production and Maintain CD8+ T Cell Suppression during Chronic Liver Disease. Hepatology.

[239] Tang Q., Henriksen K.J., Bi M., Finger E.B., Szot G., Ye J., Masteller E.L., McDevitt H., Bonyhadi M., Bluestone J.A. (2004). In Vitro–expanded Antigen-specific Regulatory T Cells Suppress Autoimmune Diabetes. J. Exp. Med..

[240] Longhi M.S., Hussain M.J., Kwok W.W., Mieli-Vergani G., Ma Y., Vergani D. (2011). Autoantigen-specific regulatory T cells, a potential tool for immune-tolerance reconstitution in type-2 autoimmune hepatitis. Hepatology.

[241] Liang M., Liwen Z., Juan D., Yun Z., Yanbo D., Jianping C. (2020). Dysregulated TFR and TFH cells correlate with B-cell differentiation and antibody production in autoimmune hepatitis. J. Cell. Mol. Med..

[242] Morita M., Fujino M., Jiang G., Kitazawa Y., Xie L., Azuma M., Yagita H., Nagao S., Sugioka A., Kurosawa Y. (2010). PD-1/B7-H1 Interaction Contribute to the Spontaneous Acceptance of Mouse Liver Allograft. Am. J. Transplant..

[243] Kido M., Watanabe N., Okazaki T., Akamatsu T., Tanaka J., Saga K., Nishio A., Honjo T., Chiba T. (2008). Fatal Autoimmune Hepatitis Induced by Concurrent Loss of Naturally Arising Regulatory T Cells and PD-1-Mediated Signaling. Gastroenterology.

[244] Aoki N., Kido M., Iwamoto S., Nishiura H., Maruoka R., Tanaka J., Watanabe T., Tanaka Y., Okazaki T., Chiba T. (2011). Dysregulated Generation of Follicular Helper T Cells in the Spleen Triggers Fatal Autoimmune Hepatitis in Mice. Gastroenterology.

[245] Jeffery H.C., Braitch M.K., Bagnall C., Hodson J., Jeffery L.E., Wawman R.E., Wong L.L., Birtwistle J., Bartlett H., Lohse A.W. (2018). Changes in natural killer cells and exhausted memory regulatory T Cells with corticosteroid therapy in acute autoimmune hepatitis. Hepatol. Commun..

[246] Lapierre P., Béland K., Yang R., Alvarez F. (2013). Adoptive transfer of ex vivo expanded regulatory T cells in an autoimmune hepatitis murine model restores peripheral tolerance. Hepatology.

[247] Oo Y.H., Ackrill S., Cole R., Jenkins L., Anderson P., Jeffery H.C., Jones N., Jeffery L.E., Lutz P., Wawman R.E. (2019). Liver homing of clinical grade Tregs after therapeutic infusion in patients with autoimmune hepatitis. JHEP Rep..

[248] Buitrago-Molina L.E., Pietrek J., Noyan F., Schlue J., Manns M.P., Wedemeyer H., Hardtke-Wolenski M., Jaeckel E. (2021). Treg-specific IL-2 therapy can reestablish intrahepatic immune regulation in autoimmune hepatitis. J. Autoimmun..

[249] Marceau G., Yang R., Lapierre P., Béland K., Alvarez F. (2015). Low-dose anti-CD3 antibody induces remission of active auto-immune hepatitis in xenoimmunized mic. Liver Int..

[250] Ichiki Y., Aoki C.A., Bowlus C., Shimoda S., Ishibashi H., Gershwin M.E. (2005). T cell immunity in autoimmune hepatitis. Autoimmun. Rev..

[251] Hashimoto E., Lindor K.D., Homburger H.A., Dickson E.R., Czaja A.J., Wiesner R.H., Ludwig J. (1993). Immunohistochemical Characterization of Hepatic Lymphocytes in Primary Biliary Cirrhosis in Comparison With Primary Sclerosing Cholangitis and Autoimmune Chronic Active Hepatitis. Mayo Clin. Proc..

[252] Tordjmann T., Soulie A., Guettier C., Schmidt M., Berthou C., Beaugrand M., Sasportes M. (2008). Perforin and granzyme B lytic protein expression during chronic viral and autoimmune hepatitis. Liver Int..

[253] Longhi M.S., Hussain M.J., Bogdanos D.P., Quaglia A., Mieli-Vergani G., Ma Y., Vergani D. (2007). Cytochrome P450IID6-specific CD8 T cell immune responses mirror disease activity in autoimmune hepatitis type 2. Hepatology.

[254] Holz L.E., Benseler V., Vo M., McGuffog C., van Rooijen N., McCaughan G.W., Bowen D.G., Bertolino P. (2012). Naïve CD8 T cell activation by liver bone marrow-derived cells leads to a “neglected” IL-2low Bimhigh phenotype, poor CTL function and cell death. J. Hepatol..

[255] Rajoriya N., Fergusson J.R., Leithead J.A., Klenerman P. (2014). Gamma Delta T-Lymphocytes in Hepatitis C and Chronic Liver Disease. Front. Immunol..

[256] Wen L., Peakman M., Mieli-Vergani G., Vergani D. (1992). Elevation of Activated Gamma Delta T Cell Receptor Bearing T Lymphocytes in Patients with Autoimmune Chronic Liver Disease. Clin. Exp. Immunol..

[257] Kasper H.U., Ligum D., Cucus J., Stippel D.L., Dienes H.P., Drebber U. (2009). Liver Distribution of Gammadelta-T-Cells in Patients with Chronic Hepatitis of Different Etiology. APMIS.

[258] Stinissen P., Vandevyver C., Medaer R., Vandegaer L., Nies J., Tuyls L., Hafler D.A., Raus J., Zhang J. (1995). Increased Frequency of Gamma Delta T Cells in Cerebrospinal Fluid and Peripheral Blood of Patients with Multiple Sclerosis. Reactivity, Cytotoxicity, and T Cell Receptor V Gene Rearrangements. J. Immunol..

[259] Bank I., Duvdevani M., Livneh A. (2003). Expansion of Gammadelta T-Cells in Behçet’s Disease: Role of Disease Activity and Microbial Flora in Oral Ulcers. J. Lab. Clin. Med..

[260] Marquardt N., Béziat V., Nyström S., Hengst J., Ivarsson M.A., Kekäläinen E., Johansson H., Mjösberg J., Westgren M., Lankisch T.O. (2015). Cutting Edge: Identification and Characterization of Human Intrahepatic CD49a+ NK Cells. J. Immunol..

[261] Hudspeth K., Donadon M., Cimino M., Pontarini E., Tentorio P., Preti M., Hong M., Bertoletti A., Bicciato S., Invernizzi P. (2016). Human liver-resident CD56(bright)/CD16(neg) NK cells are retained within hepatic sinusoids via the engagement of CCR5 and CXCR6 pathways. J. Autoimmun..

[262] Tian Z., Chen Y., Gao B. (2013). Natural killer cells in liver disease. Hepatology.

[263] Dong Z., Wei H., Sun R., Hu Z., Gao B., Tian Z. (2004). Involvement of natural killer cells in PolyI:C-induced liver injury. J. Hepatol..

[264] Grant A.J., Goddard S., Ahmed-Choudhury J., Reynolds G., Jackson D.G., Briskin M., Wu L., Hübscher S.G., Adams D.H. (2002). Hepatic Expression of Secondary Lymphoid Chemokine (CCL21) Promotes the Development of Portal-Associated Lymphoid Tissue in Chronic Inflammatory Liver Disease. Am. J. Pathol..

[265] Taubert R., Hardtke-Wolenski M., Noyan F., Wilms A., Baumann A.K., Schlue J., Olek S., Falk C.S., Manns M.P., Jaeckel E. (2014). Intrahepatic regulatory T cells in autoimmune hepatitis are associated with treatment response and depleted with current therapies. J. Hepatol..

[266] Burak K.W., Swain M.G., Santodomingo-Garzon T., Lee S.S., Urbanski S.J., Aspinall A., Coffin C.S., Myers R.P. (2013). Rituximab for the Treatment of Patients with Autoimmune Hepatitis Who are Refractory or Intolerant to Standard Therapy. Can. J. Gastroenterol..

[267] Than N.N., Hodson J., Schmidt-Martin D., Taubert R., Wawman R.E., Botter M., Gautam N., Bock K., Jones R., Appanna G.D. (2019). Efficacy of rituximab in difficult-to-manage autoimmune hepatitis: Results from the In-ternational Autoimmune Hepatitis Group. JHEP Rep..

[268] Buitrago-Molina L., Dywicki J., Noyan F., Schepergerdes L., Pietrek J., Lieber M., Schlue J., Manns M., Wedemeyer H., Jaeckel E. (2021). Anti-CD20 Therapy Alters the Protein Signature in Experimental Murine AIH, but Not Exclusively towards Regeneration. Cells.

[269] Arvaniti P., Giannoulis G., Gabeta S., Zachou K., Koukoulis G.K., Dalekos G.N. (2020). Belimumab is a promising third-line treatment option for refractory autoimmune hepatitis. JHEP Rep..

[270] Eibl M. (1985). Immunological Consequences of Splenectomy. Prog. Pediatric. Surg..

[271] Karakantza M., Theodorou G.L., Mouzaki A., Theodori E., Vagianos C., Maniatis A. (2004). In Vitro Study of the Long-Term Effects of Post-Traumatic Splenectomy on Cellular Immunity. Scand. J. Immunol..

[272] Kim M.T., Harty J.T. (2014). Splenectomy Alters Distribution and Turnover but not Numbers or Protective Capacity of de novo Generated Memory CD8T-Cells. Front. Immunol..

[273] Kurokohchi K., Masaki T., Himoto T., Deguchi A., Nakai S., Morishita A., Yoneyama H., Kimura Y., Watanabe S., Kuriyama S. (2006). Usefulness of liver infiltrating CD86-positive mononuclear cells for diagnosis of autoimmune hepatitis. World J. Gastroenterol..

[274] Clemente-Casares X., Blanco J., Ambalavanan P., Yamanouchi J., Singha S., Fandos C., Tsai S., Wang J., Garabatos N., Izquierdo C. (2016). Expanding antigen-specific regulatory networks to treat autoimmunity. Nature.

[275] Ellebrecht C.T., Bhoj V.G., Nace A., Choi E.J., Mao X., Cho M.J., Di Zenzo G., Lanzavecchia A., Seykora J.T., Cotsarelis G. (2016). Reengineering chimeric antigen receptor T cells for targeted therapy of autoimmune disease. Science.

[276] Lied G.A., Berstad A. (2010). Functional and Clinical Aspects of the B-Cell-Activating Factor (BAFF): A Narrative Review. Scand. J. Immunol..

[277] Liberal R., Mieli-Vergani G., Vergani D. (2016). Contemporary issues and future directions in autoimmune hepatitis. Expert Rev. Gastroenterol. Hepatol..

[278] Pardoll D.M. (2012). The blockade of immune checkpoints in cancer immunotherapy. Nat. Rev. Cancer.

